# Indoxyl sulfate reduces I_to,f_ by activating ROS/MAPK and NF-**κ**B signaling pathways

**DOI:** 10.1172/jci.insight.145475

**Published:** 2022-02-08

**Authors:** Jing Yang, Hongxia Li, Chi Zhang, Yafeng Zhou

**Affiliations:** 1 Department of Cardiology, The First Affiliated Hospital of Soochow University, Suzhou, Jiangsu, China.; 2 Department of Cardiology, Dushu Lake Hospital Affiliated to Soochow University, Suzhou, Jiangsu, China.

**Keywords:** Cardiology, Arrhythmias, Ion channels, Molecular biology

## Abstract

There is a high prevalence of ventricular arrhythmias related to sudden cardiac death in patients with chronic kidney disease (CKD). To explored the possible mechanism of CKD-related ventricular arrhythmias, a CKD rat model was created, and indoxyl sulfate (IS) was further used in vivo and in vitro. This project used the following methods: patch clamp, electrocardiogram, and some molecular biology experimental techniques. IS was found to be significantly elevated in the serum of CKD rats. Interestingly, the expression levels of the fast transient outward potassium current–related (I_to,f_-related) proteins (Kv4.2, Kv4.3, and KChIP2) in the heart of CKD rats and rats treated with IS decreased. IS dose-dependently reduced I_to,f_ density, accompanied by the decreases in Kv4.2, Kv4.3, and KChIP2 proteins in vitro. IS also prolonged the action potential duration and QT interval, and paroxysmal ventricular tachycardia could be induced by IS. In-depth studies have shown that ROS/p38MAPK, ROS–p44/42 MAPK, and NF-κB signaling pathways play key roles in the reduction of I_to,f_ density and I_to,f_-related proteins caused by IS. These data suggest that IS reduces I_to,f_-related proteins and I_to,f_ density by activating ROS/MAPK and NF-κB signaling pathways, and the action potential duration and QT interval are subsequently prolonged, which contributes to increasing the susceptibility to arrhythmia in CKD.

## Graphical abstract

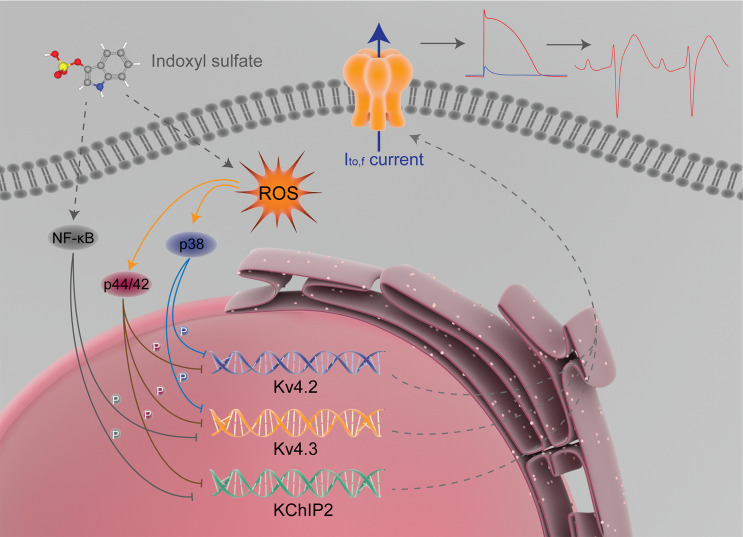

## Introduction

Chronic kidney disease (CKD) affects 5%–7% of the global population and is associated with a 10-fold increase in mortality from cardiovascular disease (1). According to the US Kidney Data System (USRDS), the main cause of death is related to arrhythmia in CKD patients undergoing dialysis (2). Some studies have shown that patients with dialysis have a 19%–72% prevalence of ventricular arrhythmias based on results from 24-hour electrocardiogram (ECG) monitoring (Holter monitor) (3–6). However, the mechanism of ventricular arrhythmias in patients with CKD remains ambiguous.

The deterioration of renal function leads to the gradual retention of a large number of uremic toxins, which are normally excreted by healthy kidneys (7). Uremic toxins can be divided into small water-soluble molecules (molecular weight [MW] < 500 Da), middle molecular (MW > 500 Da), and protein-bound molecules according to the physicochemical characteristics that affect the uremic toxin clearance rate during dialysis (8). The last category is characterized by limited dialysis removal due to the high molecular weight of the protein complex, which complicates the movement on the dialysis membrane (9). Indoxyl sulfate (IS), a typical protein-binding uremic toxin, is easily accumulated in the blood of CKD patients (10, 11). A study shows that serum IS level is a valuable indicator to predict cardiovascular events in patients with advanced CKD (12). High IS level in plasma is associated with the first heart-failure event (13). In addition, patients with elevated serum IS levels have a higher recurrence rate of atrial fibrillation after successful catheter ablation, which can be used as an important predictor of atrial fibrillation recurrence (14). Serum IS levels are also associated with prolongation of the QT interval, which is a dangerous sign of ventricular arrhythmias (15). Therefore, IS may be involved in the occurrence and development of arrhythmia in CKD patients.

The fast transient outward potassium current (I_to,f_) is composed of voltage-gated α pore-forming subunits (Kv4.3 in humans; Kv4.2 and Kv4.3 in rodents) and the accessory β subunit KChIP2 (16). There is a clear transmural gradient in the expression of the subunits that make up I_to,f_, which leads to the heterogeneous distribution of I_to,f_ in the ventricle (17). I_to,f_ contributes to the early repolarization period of the action potential (AP) and can affect the AP duration (APD) (18). It plays a key role in myocardial excitation-contraction coupling through its influence on L-type Ca^2+^ current (18, 19). In particular, changes in I_to,f_ in the human heart are closely related to the development of Brugada syndrome (20).

IS has the ability to induce oxidative stress in the proximal tubule cells of the kidney (21) and can reduce the superoxide scavenging activity in the kidneys of CKD rats (22). A study shows that IS upregulates the expression of renal intercellular adhesion molecule 1 through the production of reactive oxygen species (ROS) such as superoxide and the activation of NF-κB in proximal tubular cells (23). It also regulates angiogenesis and myotube atrophy through the production of ROS (24, 25). In addition, IS downregulates the expression of nuclear factor (erythroid-derived 2)-like 2 (Nrf2) and Klotho in the kidney by activating NF-κB (26, 27). IS has the potential to play a key role in adverse cardiac remodeling by activating p38 MAPK, p42/44 MAPK, and NF-κB pathways (28). Some studies show that IS stimulates the bulbospinal neurons in the rostral ventrolateral medulla (29) and increases pulmonary vein burst firings and delayed after depolarizations by producing oxidative stress (30). However, the effect of IS on I_to,f_ and its role in the pathogenesis of CKD-related arrhythmia are still unclear.

Therefore, we explored the possible mechanism of IS regulating I_to,f_ and its role in CKD-related ventricular arrhythmias. We found that IS reduced I_to,f_ by activating ROS/MAPK and NF-κB signaling pathways, and the reduced I_to,f_ caused by IS contributed to CKD-related ventricular arrhythmias.

## Results

### I_to,f_-related proteins were downregulated in CKD rats with a high level of IS.

**Figure 1:**
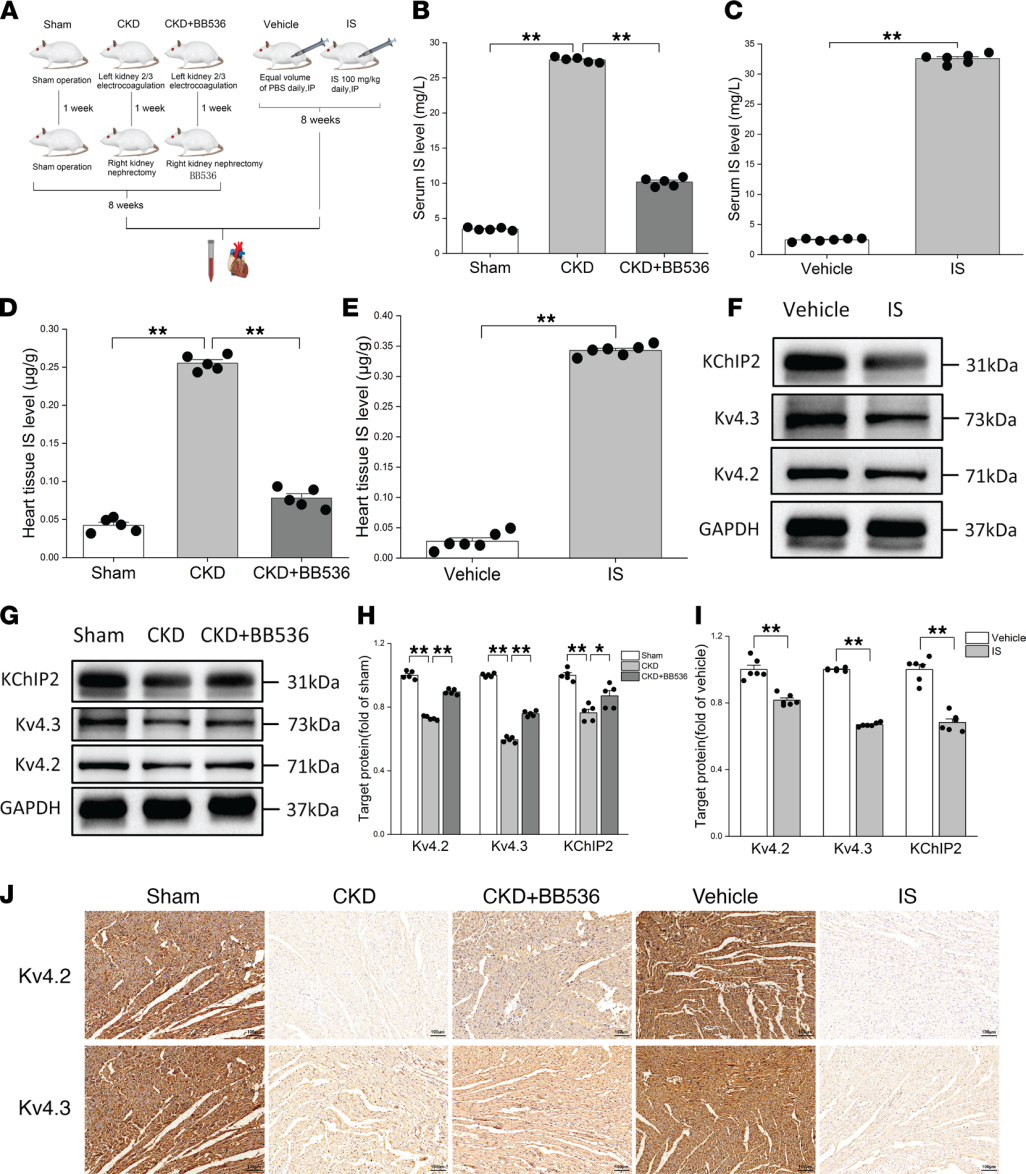
I_to,f_-related proteins were downregulated in CKD rats with a high levels of IS. (**A**) Flow chart of animal experiments. (**B** and **D**) Determinations of IS levels in serum (**B**) and heart tissues (**D**) at 8 weeks after right nephrectomy (*n* = 5 per group). (**C** and **E**) Measurements of IS levels in serum (**C**) and heart tissues (**E**) after 8 weeks of IS treatment (*n* = 6 per group). (**F** and **I**) Representative immunoblots (**F**) and average data (**I**) of Kv4.2, Kv4.3, and KChIP2 proteins in vehicle and IS treatment groups (*n* = 6 per group). (**G** and **H**) Representative immunoblots (**G**) and average data (**H**) of Kv4.2, Kv4.3, and KChIP2 proteins in sham, CKD, and CKD plus BB536 groups (*n* = 5 per group). (**J**) Representative IHC images of Kv4.2 and Kv4.3 proteins in rats. Scale bar: 100 μm. Data are presented as mean ± SEM. Statistical analysis was performed using 2-tailed Student’s *t* test (**C**, **E**, and **I**) and 1-way ANOVA followed by Bonferroni post hoc test (**B**, **D**, and **H**). *P < 0.05, **P < 0.01.

In order to explore the changes of I_to,f_-related proteins in the heart of a CKD rat model, a 5/6 nephrectomy CKD rat model was created (Figure 1A). After 8 weeks of model creation, serum creatinine and urea nitrogen in CKD rats were significantly increased (Supplemental Figure 1, A and B; supplemental material available online with this article; https://doi.org/10.1172/jci.insight.145475DS1) compared with sham group. Interestingly, IS in heart tissue and serum of CKD rats were also significantly increased (Figure 1, B and D). We then further investigated the changes of I_to,f_-related proteins in CKD rats using Western blot and IHC. Surprisingly, the results of Western blot showed that I_to,f_-related proteins (Kv4.2, Kv4.3, and KChIP2) in the heart of CKD rats were significantly lower than those in the sham group (Figure 1, G and H), and the results of IHC also showed that Kv4.2 and Kv4.3 were downregulated in the heart of CKD rats compared with sham group (Figure 1J). However, BB536, a bifidobacterium longum strain, effectively reduced IS in heart tissue and serum of CKD rats (Figure 1, B and D), and this effect was similar to a previous study (31). It also partially reversed the downregulation of I_to,f_-related proteins in CKD rats. This suggests that the downregulation of I_to,f_-related proteins may be due to the high level of IS in heart tissue in CKD rat models.

To test the hypothesis in vivo, normal Wistar rats were treated with IS for 8 weeks, after that, we found that the IS levels in the heart tissues and serum of rats treated with IS were significantly higher than those in the vehicle group (Figure 1, C and E). Importantly, I_to,f_-related proteins were downregulated in the IS treatment group (Figure 1, F, I, and J). These findings indicate that IS plays a key role in the downregulation of I_to,f_-related proteins in CKD, which may lead to the change of I_to,f_.

### IS reduced I_to,f_-related protein expression levels and current densities in vitro.

**Figure 2:**
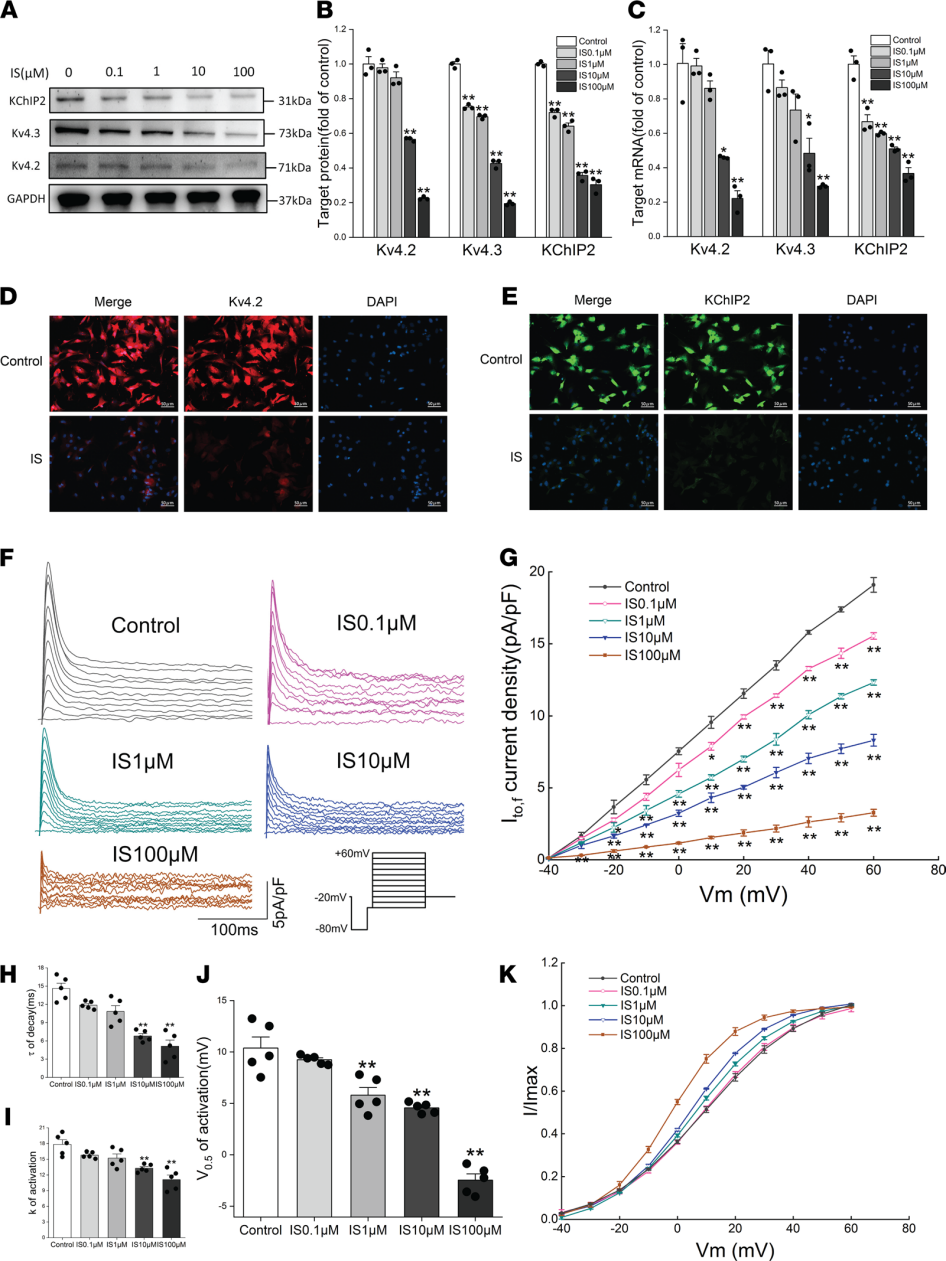
IS reduced I_to,f_-related protein expression levels and current densities in vitro. (**A** and **B**) Representative immunoblots (**A**) and average data (**B**) of Kv4.2, Kv4.3, and KChIP2 proteins in NRVMs treated with different concentrations of IS (*n* =3 per group). (**C**) Relative mRNA expressions for Kv4.2, Kv4.3, and KChIP2 in NRVMs treated with different concentrations of IS (*n* = 3 per group). (**D** and **E**) Representative immunofluorescence images of Kv4.2 (**D**) and KChIP2 (**E**) proteins in NRVMs. Scale bar: 50 μm. (**F** and **G**) Representative I_to,f_ traces (**F**) and average I_to,f_ densities (peak minus steady state) versus membrane potentials (**G**) in NRVMs treated with different concentrations of IS (*n* = 5 per group).The inset in **F** shows the voltage-clamp protocol. (**H**) Average time constants (τ) of decay of I_to,f_ at +60 mV in NRVMs (*n* = 5 per group). (**I** and **J**) Average values of constants (k) of activation (**I**) and half-maximal voltage of activation(V_0.5_, act) (**J**) in NRVMs (*n* = 5 per group). (**K**) Voltage-dependent activation curves of I_to,f_ in NRVMs (*n* = 5 per group). Data are presented as mean ± SEM. Statistical analysis was performed using 1-way ANOVA followed by Bonferroni post hoc test. *P < 0.05 versus control, **P < 0.01 versus control.

The effects of IS on I_to,f_-related proteins and current densities were investigated in cultured neonatal rat ventricular myocytes (NRVMs) with or without IS treatment. The purity of NRVMs was first identified using immunofluorescence, and the results showed that nearly all cardiomycytes were positive for cardiac troponin I (cTNI) (Supplemental Figure 1D), which suggests that high-purity NRVMs were used throughout the experiment. The NRVMs were then treated with different concentrations of IS for 24 hours, and the cell viability was not affected (Supplemental Figure 1C). However, it was found that IS treatment remarkably reduced the protein and mRNA expression levels of Kv4.2, Kv4.3, and KChIP2 in a dose-dependent manner (Figure 2, A–C). Similarly, the results of immunofluorescence also showed that the protein expression levels of Kv4.2 and KChIP2 were downregulated in NRVMs treated with 10 μM IS (Figure 2, D and E). We then recorded I_to,f_ in NRVMs using a whole-cell voltage-clamp technique. As expected, the peak current densities of I_to,f_ was significantly decreased in the IS treatment group with different concentrations compared with that in the control group (Figure 2, F and G). It is worth mentioning that the peak current density values of I_to,f_ decreased greatly from the treatment of IS with a concentration of 10 μM.

The gating kinetic characteristics of I_to,f_ were further studied under different concentrations of IS treatment. We used the Boltzmann equation to fit the activation curve under various command voltages, and we found that the voltages at half-maximum activation (V_0.5_, act) at 1, 10, and 100 μM were significantly lower than those in the control group (Figure 2, J and K); the k constant of activation at 10 and 100 μM was significantly lower than that in the control group (Figure 2, I and K). The time constant (τ) of decay was estimated by a monoexponential function of the currents recorded at +60 mV. The results showed that the τ of decay were significantly reduced in the IS treatment group with concentrations of 10 and 100 μM compared with the control group (Figure 2H). These results indicated that IS reduced the I_to,f_ densities by downregulating the expression of Kv4.2, Kv4.3, and KChIP2; it also accelerated the activation and decay of I_to,f_ at effective concentrations. Importantly, this provides a potential mechanism for the susceptibility of IS-induced ventricular arrhythmia in CKD.

### IS prolonged APD of NRVMs and increased the susceptibility to ventricular arrhythmia in CKD rats.

**Figure 3:**
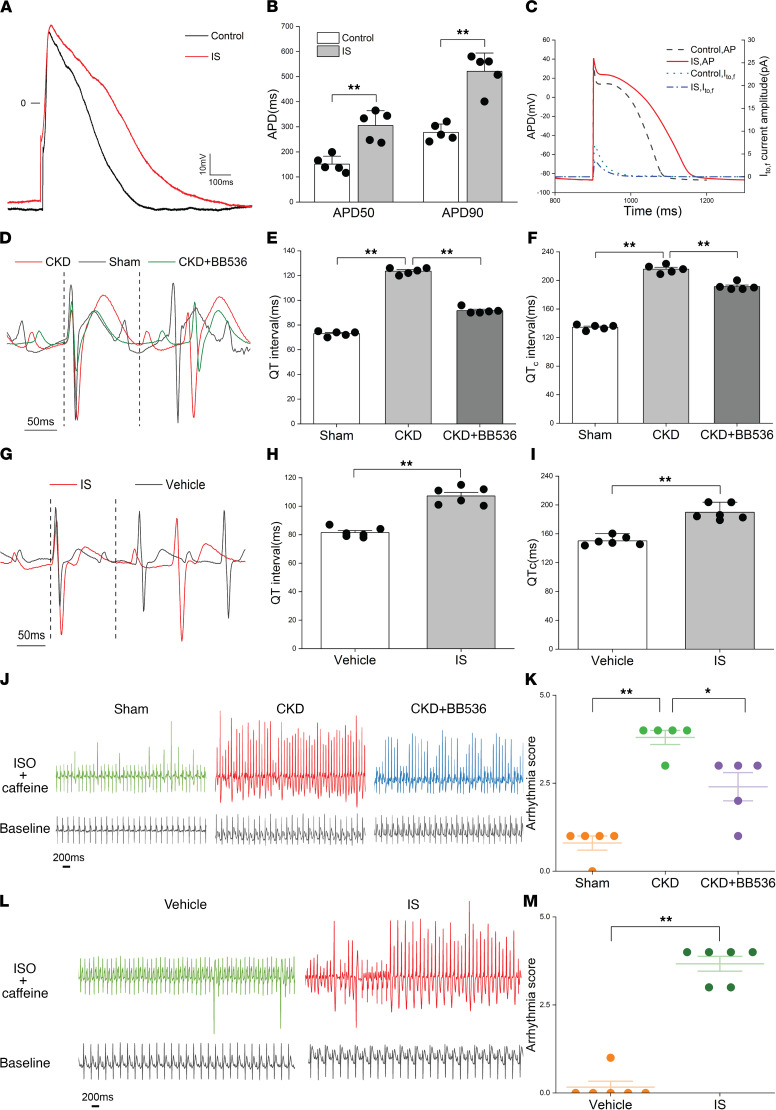
IS prolonged APD of NRVMs and increased the susceptibility to ventricular arrhythmia in CKD rats. (**A** and **B**) Representative AP traces (**A**) and average values of APD_50_ and APD_90_ in NRVMs treated with or without 10μM IS (*n* = 5 per group). (**C**) Computational models of AP in control and IS treatment groups. (**D**–**F**) Representative electrocardiograms from a lead II (**D**), QT intervals, (**E**) and corrected QT intervals (**F**) in sham, CKD, and CKD plus BB536 groups (*n* = 5 per group). (**G**–**I**) Representative electrocardiograms from a lead II (**G**), QT intervals (**H**), and corrected QT intervals (**I**) in vehicle and IS treatment groups (*n* = 6 per group). (**J** and **K**) Representative electrocardiograms of the rats before and after the i.p. injection of isoproterenol and caffeine (**J**) and arrhythmia scores (**K**) in sham, CKD, and CKD plus BB536 groups (*n* = 5 per group). (**L** and **M**) Representative electrocardiograms of the rats before and after the i.p. injection of isoproterenol and caffeine (**L**) and arrhythmia scores (**M**) in vehicle and IS treatment groups (*n* = 6 per group). Data are presented as mean ± SEM. Statistical analysis was performed using 2-tailed Student’s *t* test (**B**, **H**, **I**, and **M**) and 1-way ANOVA followed by Bonferroni post hoc test (**E**, **F**, and **K**). *P < 0.05, **P < 0.01.

Since IS can cause the reduction of I_to,f_ density, it is necessary to clarify the effect of IS on APD. The whole-cell current-clamp technique was used to record AP in NRVMs. The results showed that IS was able to significantly prolong APD (Figure 3A), and the APD values at 50% (*P* < 0.01) and 90% (*P* < 0.01) repolarization were higher in the IS treatment NRVMs (Figure 3B). Similar results from computational modeling of AP indicated that IS prolonged the APD in the mathematical model (Figure 3C). Abnormal prolongation of APD may induce a series of arrhythmias in the CKD rats with elevated IS. To verify this, we evaluated the changes in the ECG of CKD rats and IS treatment rats. Interestingly, there were significant increases in the QT interval and corrected QT interval in CKD rats, which could be partially reversed by BB536 (Figure 3, D–F). More importantly, IS treatment rats also showed increases of the QT interval and corrected QT interval compared with the vehicle group (Figure 3, G–I), which implied that IS plays an important role in the abnormal ECG of CKD rats. The susceptibility to ventricular arrhythmia was remarkably increased in CKD rats and IS treatment rats after using isoproterenol and caffeine, and BB536 could reduce the susceptibility to ventricular arrhythmia in CKD rats (Figure 3, J and L, and Supplemental Figure 1E), which was prominently displayed by the quantified arrhythmia score (Figure 3, K and M). These data indicate that IS prolongs APD and the QT interval, and it plays a key role in increasing the susceptibility to ventricular arrhythmias in CKD rats.

### ROS production was involved in IS-induced reduction of I_to,f_-related proteins and current densities.

**Figure 4:**
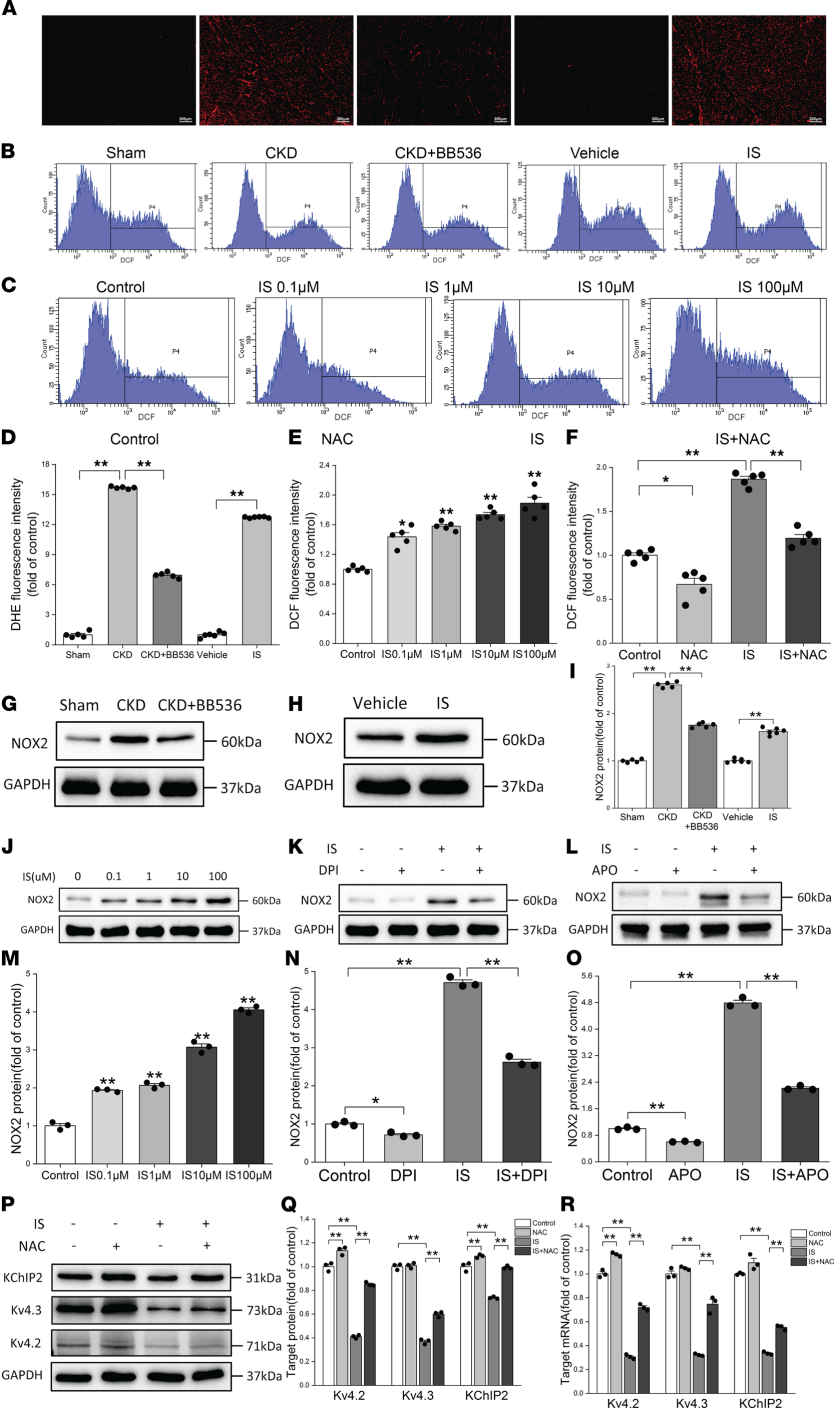
ROS production was involved in IS-induced reductions of I_to,f_-related proteins. (**A** and **D**) Measurements of ROS productions based on DHE fluorescence in rat hearts. (**A**) Representative images of DHE immunofluorescence. Scale bar: 200 μm. (**D**) Relative ROS fluorescence intensities in sham, CKD, and CKD plus BB536 groups (*n* = 5 per group), and in vehicle and IS treatment groups (*n* = 6 per group). (**B** and **E**) Measurements of ROS productions based on flow cytometry in NRVMs treated with different concentrations of IS. (**B**) Representative flow cytometric histograms in NRVMs. (**E**) Relative ROS fluorescence intensities in NRVMs (*n* = 5 per group). (**C** and **F**) NAC reversed ROS production induced by IS in NRVMs. (**C**) Representative flow cytometric histograms in 4 groups. (**F**) Relative ROS fluorescence intensities detected by flow cytometry in 4 groups (*n* = 5 per group). (**G** and **H**) Representative immunoblots of NOX2 proteins in sham, CKD, and CKD plus BB536 groups (*n* = 5 per group) (**G**), and in vehicle and IS treatment groups (*n* = 6 per group) (**H**). (**I**) Average immunoblots data of NOX2 proteins in the hearts of rats. (**J** and **M**) Representative immunoblots (**J**) and average data (**M**) of NOX2 proteins in NRVMs treated with different concentrations of IS (*n* = 3 per group). (**K** and **N**) Representative immunoblots (**K**) and average data (**N**) of NOX2 proteins in control, DPI, IS, and IS plus DPI groups (*n* = 3 per group). (**L** and **O**) Representative immunoblots (**L**) and average data (**O**) of NOX2 proteins in control, APO, IS, and IS plus APO groups (*n* = 3 per group). (**P** and **Q**) Representative immunoblots (**P**) and average data (**Q**) of Kv4.2, Kv4.3, and KChIP2 proteins in control, NAC, IS, and IS plus NAC groups (*n* = 3 per group). (**R**) Relative mRNA expressions for Kv4.2, Kv4.3, and KChIP2 in control, NAC, IS, and IS plus NAC groups (*n* = 3 per group). NRVMs in IS and IS plus NAC groups were treated with 10 μM IS. Data are presented as mean ± SEM. Statistical analysis was performed using 2-tailed Student’s *t* test (**D** and **I**) and 1-way ANOVA followed by Bonferroni post hoc test (**D**–**F**, **I**, **M**–**O**, **Q**, and **R**). *P < 0.05, **P < 0.01.

To explore the specific mechanism of IS-induced reduction of I_to,f_-related proteins, we detected the production of ROS using immunofluorescence and flow cytometry. Oxidized fluorescent probe dihydroethidium (DHE) was used to detect in situ levels of ROS in rat hearts, and peroxide-sensitive fluorescent probe 2′,7′-dichlorofluorescein diacetate (H2DCFDA) was used to detect intracellular ROS production. The results showed that ROS levels were significantly increased in CKD rats and the sham group, while BB536 treatment significantly reduced ROS levels in CKD rats (Figure 4, A and D). We also found that IS treatment significantly increased ROS levels in vivo compared with the vehicle group (Figure 4, A and D), and IS treatment dose-dependently increased ROS production in vitro (Figure 4, B and E, and Supplemental Figure 2A). IS-induced ROS production was significantly eliminated by preincubation with the ROS scavenger N-acetylcysteine (NAC) (Figure 4, C and F, and Supplemental Figure 2B).

To discover the potential source of ROS, we verified the expressions of NOX2 proteins (a type of NAD[P]H oxidase subunits that are mainly expressed in cardiomyocytes) in vivo and in vitro, and we found that the expressions of NOX2 proteins were significantly elevated in the hearts of CKD rats, which could be partially reversed by BB536 (Figure 4, G and I). The NOX2 proteins in the hearts of IS-treated rats were also found to be elevated (Figure 4, H and I). Similarly, IS could increase the expression levels of NOX2 proteins in NRVMs in a dose-dependent manner (Figure 4, J and M). Both diphenyleneiodonium chloride (DPI) and apocynin (APO), which were effective NADPH oxidase inhibitors, partially reversed the effects of IS (Figure 4, K, L, N, and O). This suggests that NAD(P)H oxidase is involved in the production of ROS in IS-treated rats and cardiomyocytes.

**Figure 5:**
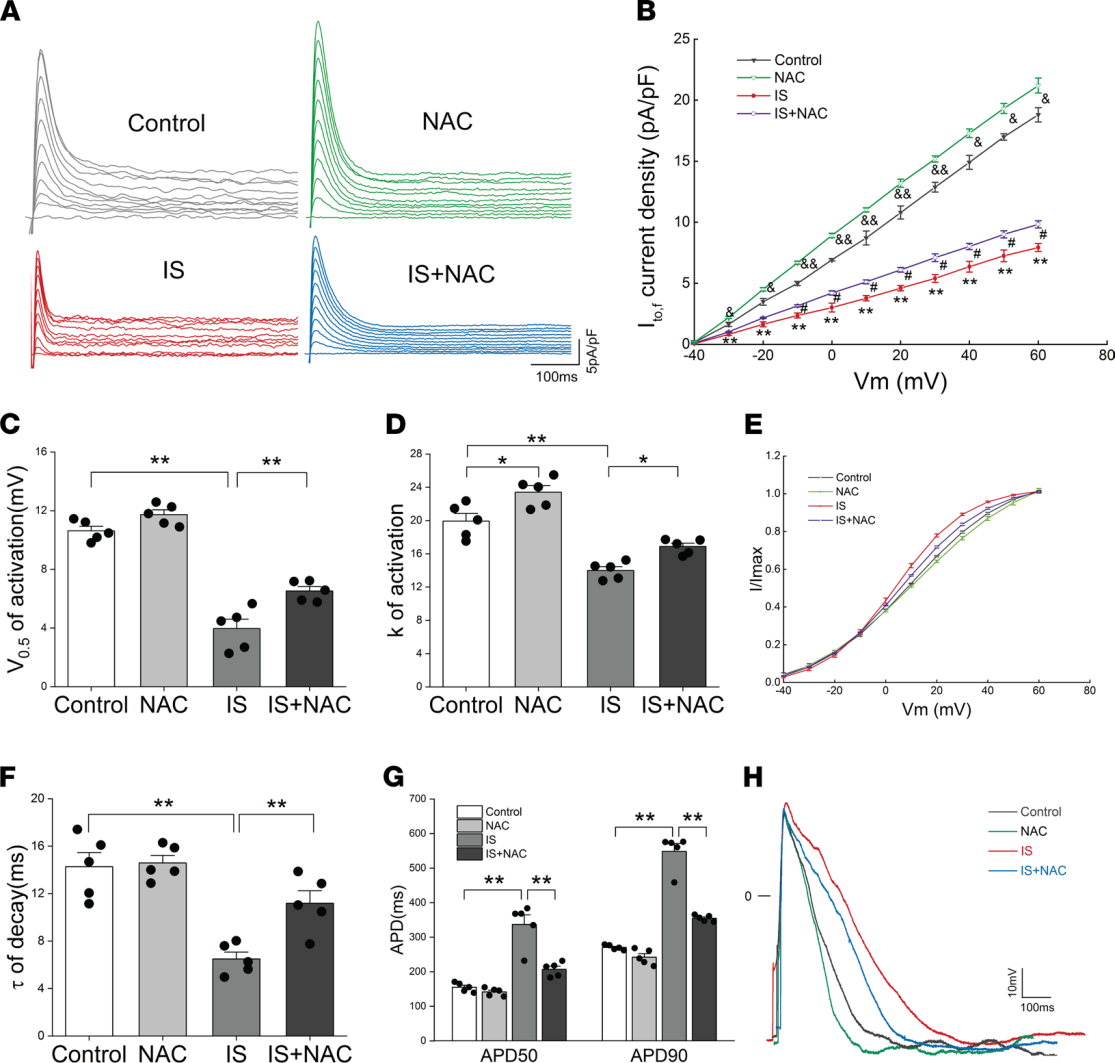
IS reduced I_to,f_ densities by generating ROS. (**A** and **B**) Representative I_to,f_ traces (**A**) and average I_to,f_ densities (peak minus steady state) versus membrane potentials (**B**) in control, NAC, IS, and IS plus NAC groups (*n* = 5 per group). ^&^*P* < 0.05, ^&&^*P* < 0.01 NAC versus Control. ^#^*P* < 0.05 IS plus NAC versus IS. **P < 0.01 IS versus control. (**C** and **D**) Average values of half-maximal voltage of activation (V_0.5_, act) (**C**) and constants(k) of activation (**D**) in control, NAC, IS, and IS plus NAC groups (*n* = 5 per group). *P < 0.05, **P < 0.01. (**E**) Voltage-dependent activation curves of I_to,f_ in 4 groups (*n* = 5 per group). (**F**) Average time constants (τ) of decay of I_to,f_ at +60 mV in 4 groups (*n* = 5 per group). **P < 0.01. (**G**) Average values of APD_50_ and APD_90_ in control, NAC, IS, and IS plus NAC groups (*n* = 5 per group). **P < 0.01. (**H**) Representative AP traces from 4 groups. NRVMs in IS and IS plus NAC groups were treated with 10 μM IS. NAC, N-acetylcysteine. Data are presented as mean ± SEM. Statistical analysis was performed using 1-way ANOVA, followed by Bonferroni post hoc test.

In addition, the cell viability was not affected (Supplemental Figure 1C). We further investigated whether IS-induced reduction of I_to,f_-related proteins was regulated by ROS; surprisingly, NAC significantly ameliorated IS-induced reductions in Kv4.2, Kv4.3, and KChIP2 proteins and genes (Figure 4, P–R); immunofluorescence showed similar results for the changes of Kv4.2 and KChIP2 protein (Supplemental Figure 2, C and D). It suggests that ROS production is involved in IS-induced reduction of I_to,f_-related proteins. Subsequently, we verified the effect of NAC on I_to,f_ density and gating kinetic characteristics. The results showed that NAC significantly eliminated the reduction of I_to,f_ density induced by IS at voltages from –30 mV to +60 mV (Figure 5, A and B), and it significantly slowed the activation and decay of I_to,f_, which could be confirmed by the increased V_0.5_ of activation, k of activation, and τ of decay in the IS plus NAC group (Figure 5, C–F). The effect of NAC on the AP of NRVMs was further analyzed, and we observed that NAC remarkably shortened the prolonged APD caused by IS at 50% and 90% repolarization (Figure 5, G and H). These findings imply that ROS can reduce I_to,f_ density by downregulating I_to,f_-related proteins and can further extend the APD.

### p38 MAPK and p44/42 MAPK were the main signaling pathways downstream of ROS in IS-treated rats and NRVMs.

**Figure 6:**
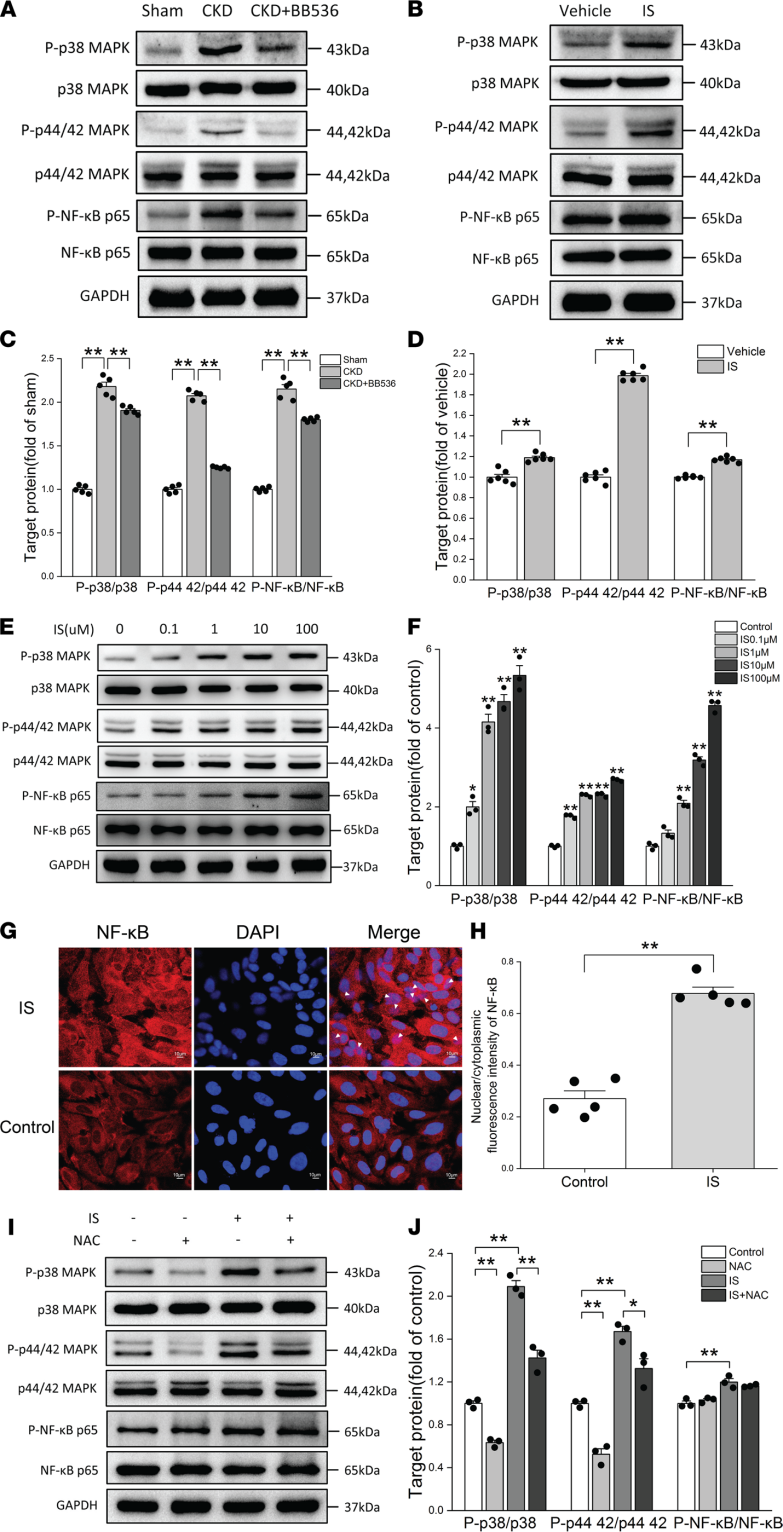
p38 MAPK and p44/42 MAPK were the main signaling pathways downstream of ROS in IS-treated rats and NRVMs. (**A** and **B**) Representative immunoblots of phosphorylated p38 (P-p38) MAPK, P-p44/42 MAPK, P-NF-κB, p38 MAPK, p44/42 MAPK, and NF-κB in sham, CKD, and CKD plus BB536 groups (**A**) and in vehicle and IS treatment groups (**B**). (**C** and **D**) Average data of P-p38 MAPK/p38 MAPK, P-p44/42 MAPK–p44/42 MAPK, and P-NF-κB/NF-κB in sham, CKD, and CKD plus BB536 groups (*n* = 5 per group) (**C**) and in vehicle and IS treatment groups (*n* = 6 per group) (**D**). **P < 0.01. (**E** and **F**) Representative immunoblots of P-p38 MAPK, P-p44/42 MAPK, P-NF-κB, p38 MAPK, p44/42 MAPK, and NF-κB (**E**) and average data (**F**) of P-p38 MAPK p38 MAPK, P-p44/42 MAPK–p44/42 MAPK and P-NF-κB/NF-κB in NRVMs treated with different concentrations of IS (*n* = 3 per group,*P < 0.05 versus control, **P < 0.01 versus control). (**G**) Translocation of NF-κB–p65 from cytoplasm to nucleus measured by immunofluorescence. White arrows indicate the nuclear localization of NF-κB–p65. Scale bar: 10 μm. (**H**) Nuclear/cytoplasmic fluorescence intensities of NF-κB in control and IS groups (*n* = 5 per group). (**I** and **J**) Representative immunoblots of P-p38 MAPK, P-p44/42 MAPK, P-NF-κB, p38 MAPK, p44/42 MAPK, and NF-κB (**I**) and average data (**J**) of P-p38 MAPK/p38 MAPK, P-p44/42 MAPK–p44/42 MAPK, and P-NF-κB/NF-κB in control, NAC, IS, and IS plus NAC groups (*n* = 3 per group). NRVMs in IS and IS plus NAC groups were treated with 10 μM IS. Data are presented as mean ± SEM. Statistical analysis was performed using 2-tailed Student’s *t* test (**D** and **H**) and 1-way ANOVA, followed by Bonferroni post hoc test (**C**, **F**, and **J**). *P < 0.05, **P < 0.01.

The above data suggest that ROS production could downregulate I_to,f_-related proteins. However, there might be some downstream signaling pathways involved in this process. Previous studies have shown that p38 MAPK and p44/42 MAPK (Erk1/2) signaling pathways are mainly downstream of ROS in various pathological processes (32, 33), and the NF-κB signaling pathway can be activated by IS in the kidney (26). So we investigated the changes of p38 MAPK, p44/42 MAPK, and NF-κB signaling pathways in IS-treated rats and NRVMs. We first observed that p38 MAPK, p44/42 MAPK, and NF-κB signaling pathways were significantly activated in CKD rats, which can be partially reversed by BB536 (Figure 6, A and C). Next, we used IS to treat Wistar rats by i.p. injection for 8 weeks. Similar results showed that p38 MAPK, p44/42 MAPK, and NF-κB signaling pathways were significantly activated in IS-treated rats (Figure 6, B and D), and IS treatment dose-dependently stimulated the phosphorylation of p38 MAPK, p44/42 MAPK, and NF-κB in NRVMs (Figure 6, E and F). In particular, immunofluorescence results showed that IS could promote the translocation of NF-κB into the nucleus (Figure 6G). The nuclear/cytoplasmic fluorescence intensity of NF-κB was subsequently calculated, and it indicated that the nuclear/cytoplasmic fluorescence intensity of NF-κB in the IS group was significantly higher than that in the control group (Figure 6H). IS-induced activation of p38 MAPK and p44/42 MAPK was significantly inhibited by NAC preincubation (Figure 6, I and J), but pretreatment had no effect on the inhibition of NF-κB phosphorylation in NRVMs (Figure 6, I and J). These results suggest that p38 MAPK and p44/42 MAPK are the main signaling pathways downstream of ROS in IS-treated rats and NRVMs, and the NF-κB signaling pathway may be directly activated by IS.

### Activation of p38 MAPK, p44/42 MAPK, and NF-κB signaling pathways downregulated I_to,f_-related proteins and current densities.

**Figure 7:**
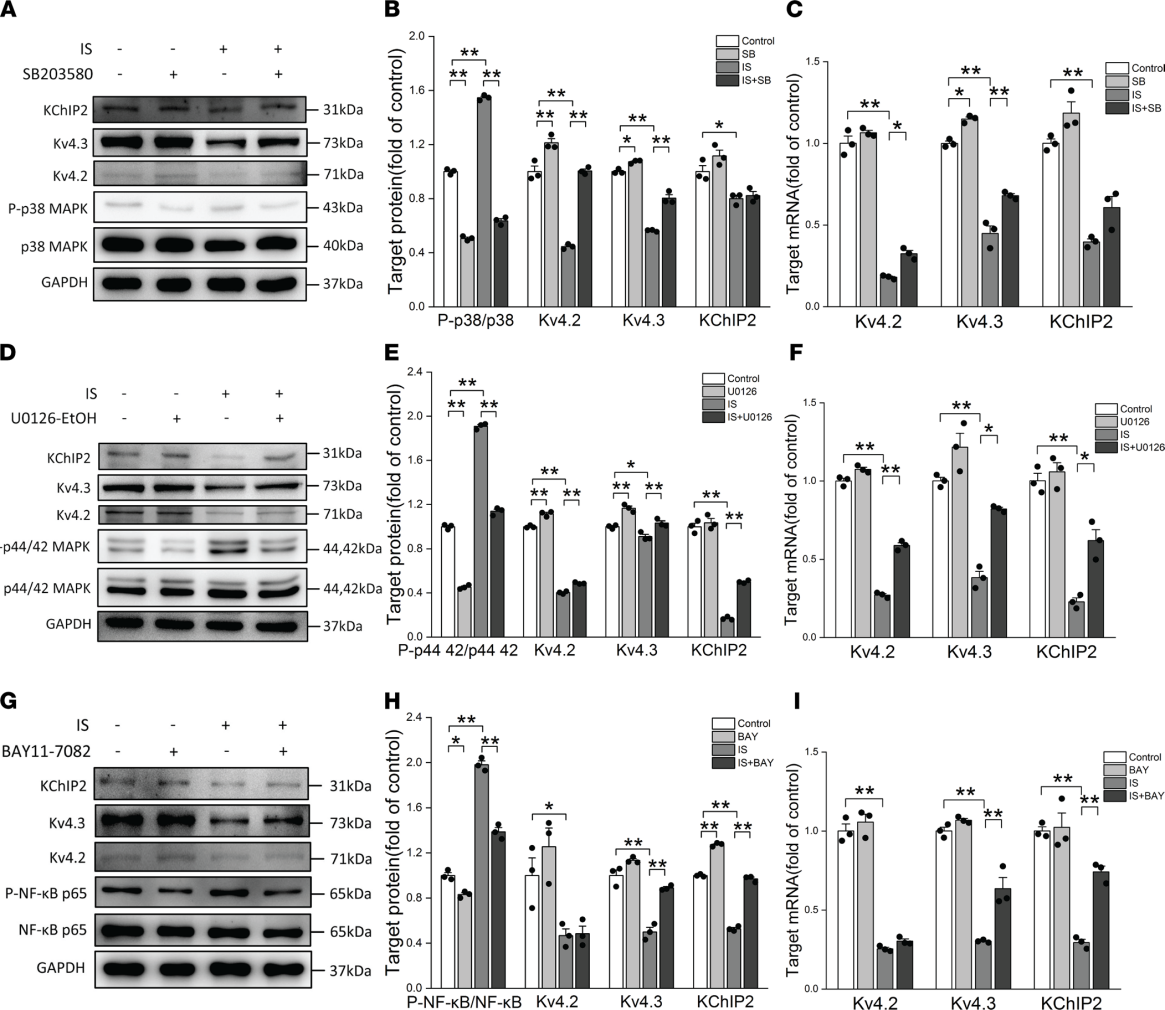
Activation of p38 MAPK, p44/42 MAPK, and NF-κB signaling pathways downregulated I_to,f_-related protein and mRNA. (**A** and **B**) Representative immunoblots (**A**) and average data (**B**) of Kv4.2, Kv4.3, KChIP2, and P-p38 MAPK in control, SB, IS, and IS plus SB groups (*n* = 3 per group). (**C**) Relative mRNA expressions for Kv4.2, Kv4.3, and KChIP2 in control, SB, IS, and IS plus SB groups (*n* = 3 per group). (**D** and **E**) Representative immunoblots (**D**) and average data (**E**) of Kv4.2, Kv4.3, KChIP2, and P-p44/42 MAPK in control, U0126, IS, and IS plus U0126 groups (*n* = 3 per group). (**F**) Relative mRNA expressions for Kv4.2, Kv4.3, and KChIP2 in control, U0126, IS, and IS plus U0126 groups (*n* = 3 per group). (**G** and **H**) Representative immunoblots (**G**) and average data (**H**) of Kv4.2, Kv4.3, KChIP2, and P-NF-κB in control, BAY, IS, and IS plus BAY groups (*n* = 3 per group). (**I**) Relative mRNA expressions for Kv4.2, Kv4.3, and KChIP2 in control, BAY, IS, and IS plus BAY groups (*n* = 3 per group). NRVMs in IS, IS plus SB, IS plus U0126, and IS plus BAY groups were treated with 10 μM IS. Data are presented as mean ± SEM. Statistical analysis was performed using 1-way ANOVA, followed by Bonferroni post hoc test. *P < 0.05, **P < 0.01.

The p38 MAPK, p44/42 MAPK, and NF-κB signaling pathways were activated in IS-treated rats and NRVMs, and p38 MAPK and p44/42 MAPK act as downstream signals of ROS. However, it was still unclear whether p38 MAPK, p44/42 MAPK, and NF-κB signaling pathways were involved in the regulation of I_to,f_-related proteins and current densities. To resolve this problem, Western blot, quantitative PCR (qPCR), and whole-cell patch clamp technology were used to detect I_to,f_-related proteins, genes, and current densities. The results show that pretreatment with SB203580, a p38MAPK inhibitor; U0126-EtOH, a p44/42 MAPK inhibitor; or BAY11-7082, an NF-κB inhibitor, significantly inhibited IS-induced activation of p38MAPK, p44/42 MAPK, and NF-κB activation, respectively (Figure 7, A, B, D, E, G, and H). Furthermore, the inhibitors did not affect cell viability (Supplemental Figure 1C). SB203580 ameliorated the inhibition effects of IS on Kv4.2 and Kv4.3 proteins and genes, which was verified by Western blot and qPCR (Figure 7, A–C), and U0126-EtOH ameliorated IS-induced reductions in Kv4.2, Kv4.3, and KChIP2 proteins and genes (Figure 7, D–F). BAY11-7082 partially reversed the inhibition effects of IS on Kv4.3 and KChIP2 proteins and genes, and this was also verified by Western blot and qPCR (Figure 7, G–I).

**Figure 8:**
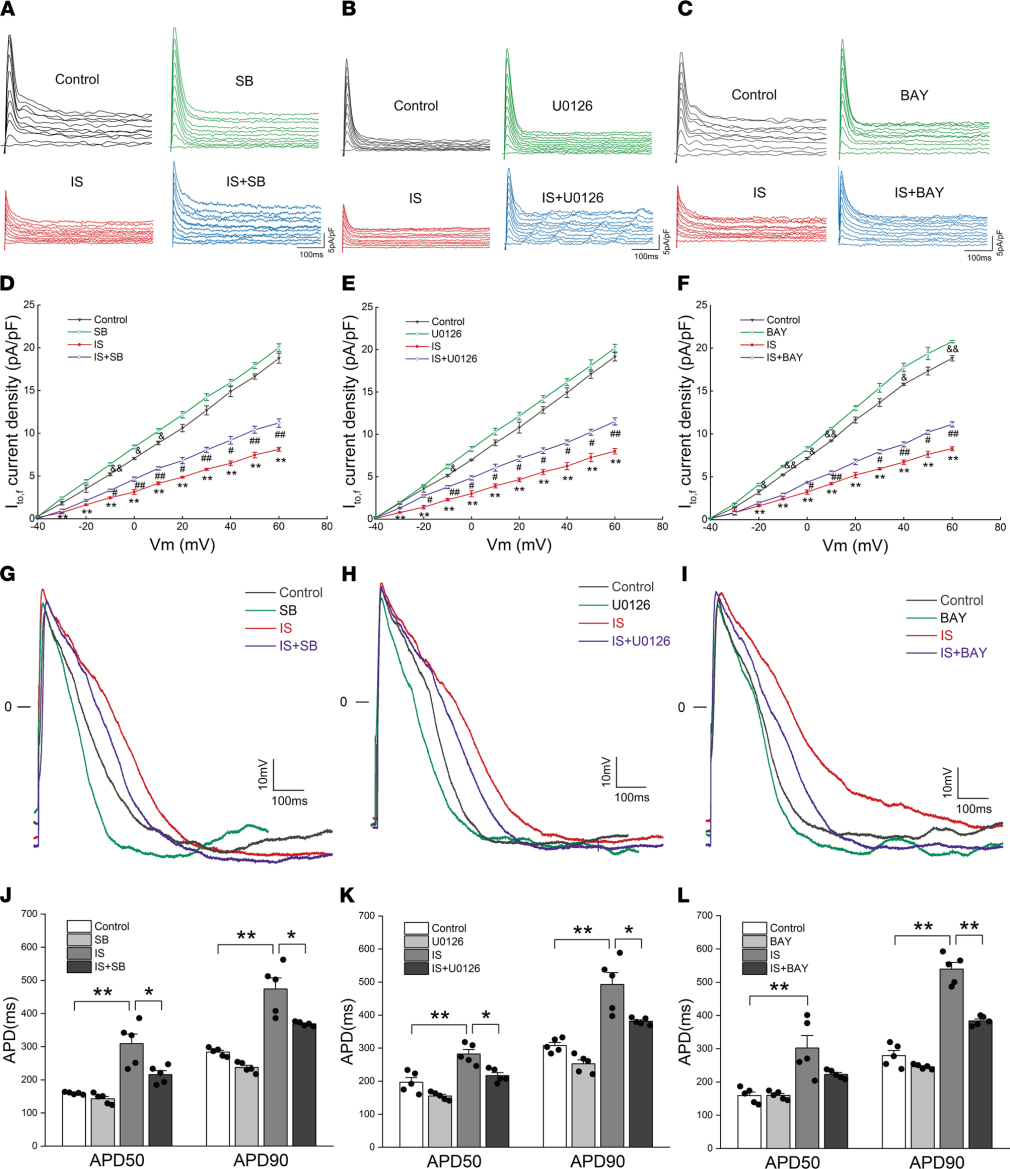
IS downregulated I_to,f_ density and prolonged APD by activating p38 MAPK, p44/42 MAPK, and NF-κB signaling pathways. (**A** and **D**) Representative I_to,f_ traces (**A**) and average I_to,f_ densities (peak minus steady state) versus membrane potentials (**D**) in control, SB, IS, and IS plus SB groups (*n* = 5 per group). ^&^*P* < 0.05, ^&&^*P* < 0.01 SB versus Control. ^#^*P* < 0.05, ^##^*P* < 0.05 IS plus SB versus IS. **P < 0.01 IS versus control. (**B** and **E**) Representative I_to,f_ traces (**B**) and average I_to,f_ densities (peak minus steady state) versus membrane potentials (**E**) in control, U0126, IS, and IS plus U0126 groups (*n* = 5 per group). ^&^*P* < 0.05 U0126 versus Control. ^#^*P* < 0.05, ^##^*P* < 0.05 IS plus U0126 versus IS. **P < 0.01 IS versus control. (**C** and **F**) Representative I_to,f_ traces (**C**) and average I_to,f_ densities (peak minus steady state) versus membrane potentials (**F**) in control, BAY, IS, and IS plus BAY groups (*n* = 5 per group). ^&^*P* < 0.05, ^&&^*P* < 0.01 BAY versus Control. ^#^*P* < 0.05, ^##^*P* < 0.05 IS plus BAY versus IS. **P < 0.01 IS versus control. (**G**–**I**) Representative AP traces from different groups treated with or without SB (**G**), U0126 (**H**), or BAY (**I**). (**J**–**L**) Average values of APD_50_ and APD_90_ in different groups treated with or without SB (**J**), U0126 (**K**), or BAY (**L**) (*n* = 5 per group). *P < 0.05, **P < 0.01. NRVMs in IS, IS plus SB, IS plus U0126, and IS plus BAY groups were treated with 10 μM IS. SB, SB203580; U0126, U0126-EtOH; BAY, BAY11-7082. Data are presented as mean ± SEM. Statistical analysis was performed using 1-way ANOVA, followed by Bonferroni post hoc test.

We further explored the effects of different signaling pathway inhibitors on I_to,f_ density, gating kinetic characteristics, and APs. The analysis results of the whole-cell voltage clamp showed that SB203580 and U0126-EtOH ameliorated the reductions of I_to,f_ densities caused by IS at voltages from –30 mV to +60 mV (Figure 8, A, B, D, and E), and BAY11-7082 partially reversed the reductions of I_to,f_ densities caused by IS at voltages from –20 mV to +60 mV (Figure 8, C and F). The changes of I_to,f_ gating kinetic characteristics were also analyzed. The results showed that SB203580 increased V_0.5_ of activation in the IS plus SB group, but it had no effect on k of activation (Supplemental Figure 3, A–C). Both U0126-EtOH and BAY11-7082 partially eliminated the IS-induced reductions of V_0.5_ and k (Supplemental Figure 3, D–I). Similarly, SB203580, U0126-EtOH, and BAY11-7082 partially reversed the reductions of τ of decay (Supplemental Figure 3, J–L). Finally, we studied the effects of these 3 signaling pathway inhibitors on the APs of NRVMs. We found that SB203580 and U0126-EtOH could significantly shorten the abnormal AP induced by IS at 50% and 90% repolarization (Figure 8, G, H, J, and K), while BAY11-7082 only significantly shortened the abnormal APD caused by IS at 90% repolarization (Figure 8, I and L). These results suggest that the activation of p38 MAPK, p44/42 MAPK, and NF-κB signaling pathways downregulate I_to,f_-related proteins and current densities, and they accelerate the activation and decay of I_to,f_ and prolong the APD. Overall, it implies that ROS/MAPK and NF-κB signaling pathways play key roles in IS-induced downregulation of I_to,f_-related proteins and current densities.

## Discussion

There is a high prevalence of ventricular arrhythmias in patients with CKD. Arrhythmia and sudden cardiac death (SCD) are the main causes of death in patients with end-stage renal disease (ESRD) (34), but the specific mechanism is poorly understood. In this study, we found that IS reduced I_to,f_ by activating ROS/MAPK and NF-κB signals. The reduced I_to,f_ could extend the APD and QT interval, which increases the susceptibility to ventricular arrhythmia in CKD. It coincides with the abnormal ECG observed in the CKD rat model after using of isoproterenol and caffeine.

The difference in I_to,f_ density is related to the diversity of AP profile in the ventricle. I_to,f_ is mainly produced by the cooperation of Kv4.2/4.3 and KChIP2 protein. And Kv4.2/4.3 is strongly expressed in the ventricles of adult rodents, dogs, and humans (35, 36). In most cases, the decrease of I_to,f_ density is accompanied by the downregulation of Kv4.2/4.3 protein (37, 38). I_to,f_ is also believed to be the cause of typical transient AP in the rodent ventricle (36). Some studies have shown that the reduction of I_to,f_ density is associated with prolonged AP in myocardial infarction and heart failure (37, 39). The reduced I_to,f_ severely suppresses or even reverses the transmural heterogeneity of the APD, which may lead to abnormal repolarization and increase the possibility of reentry arrhythmias (40, 41). Therefore, we first created a CKD rat model. IS was found to be significantly elevated in the serum and heart tissue of CKD rats. Interestingly, the expression levels of Kv4.2, Kv4.3, and KChIP2 proteins decreased in the CKD group compared with the sham group. These changes in ion channel proteins are similar to those in other disease states. Kv4.3 proteins have been verified to be downregulated in failing hearts (38, 42), while the expression levels of Kv4.2 and KChIP2 proteins decreased in the myocardial tissues of type 2 diabetic rats compared with nondiabetic controls (43). BB536, a bifidobacterium longum strain, was then used to treat CKD rats; we found that BB536 could reduce IS levels in serum and heart tissue of CKD rats, and it reversed the decreased Kv4.2, Kv4.3, and KChIP2 proteins. We suspected that IS might be involved in the process of regulating Kv4.2, Kv4.3, and KChIP2 proteins. To verify it, normal rats were treated with IS for 8 weeks, and the results showed that IS downregulated the expression levels of Kv4.2, Kv4.3, and KChIP2 proteins in the rat hearts. Because I_to,f_ is produced by Kv2, Kv3, and KCHIP2 proteins, IS has the potential to change I_to,f_ by regulating Kv4.2, Kv4.3, and KChIP2 proteins. The change of I_to,f_ can cause abnormal AP and further affect the electrical remodeling in the heart of CKD, which may explain CKD-related ventricular arrhythmia.

The reductions of Kv4.2, Kv4.3, and KChIP2 protein levels can effectively reduce the I_to,f_ density. To clarify the effect of IS on I_to,f_ density, we used IS to treat NRVMs at different concentrations for 24 hours. The results showed that IS significantly reduced Kv4.2, Kv4.3, and KChIP2 protein levels, and it dose-dependently reduced I_to,f_ density at voltages from –30 mV to +60 mV. In addition, IS accelerated the activation and decay processes of I_to,f_. This implies that IS reduces I_to,f_ density by downregulating the expressions of Kv4.2, Kv4.3, and KChIP2 proteins. The results of in-depth research indicate that IS also prolonged the APD and QT interval, which might increase the susceptibility to arrhythmia in CKD. Isoproterenol and caffeine were used to induce arrhythmia in CKD rats and IS-treated rats, paroxysmal ventricular tachycardia was observed in CKD and IS-treated rats, and arrhythmia scores in CKD and IS-treated rats were higher than the control group; BB536 could effectively relieve the abnormal electrical activity in CKD rats. This further indicates that IS-induced reduction of I_to,f_ density and acceleration of activation and decay may be involved in the formation of CKD-related ventricular arrhythmia.

ROS plays an important role in the regulation of renal function; its production can occur in the renal cortex and medulla, which has a wide range of effects, from changes in renal blood flow to fluid retention, to inflammation, fibrosis, and proteinuria (44). In addition, oxidative stress mainly including ROS is considered to be the link between inflammation and cardiovascular disease in CKD (45). Inflammation, endothelial dysfunction, aging, and calcium/phosphorus disorders contribute to the arteriosclerosis process in CKD (46). Traditional cardiovascular disease treatment methods, including HMG-CoA reductase inhibitors and angiotensin converting enzyme inhibitors, can reduce oxidative stress in the vascular system, thereby improving endothelial function and slowing cardiovascular disease progression (47, 48). IS, a representative protein-bound uremic toxin, inhibits the viability of vascular endothelial cells and promotes the proliferation of vascular smooth muscle cells by inducing ROS (49, 50). Therefore, we next investigated the role of oxidative stress in the downregulation of I_to,f_-related proteins caused by IS. We found that IS promoted the production of ROS in vivo and in vivo. NAC, a ROS scavenger, effectively reduced the production of ROS and reversed the downregulation of I_to,f_-related proteins caused by IS. And NAC also significantly reversed the reduction of I_to,f_ current density, accelerated activation and decay of I_to,f_, and extended AP in IS-treated NRVMs. It suggests that IS mainly reduces Kv4.2, Kv4.3, and KChIP2 proteins and I_to,f_ densities by promoting the production of ROS.

**Figure 9:**
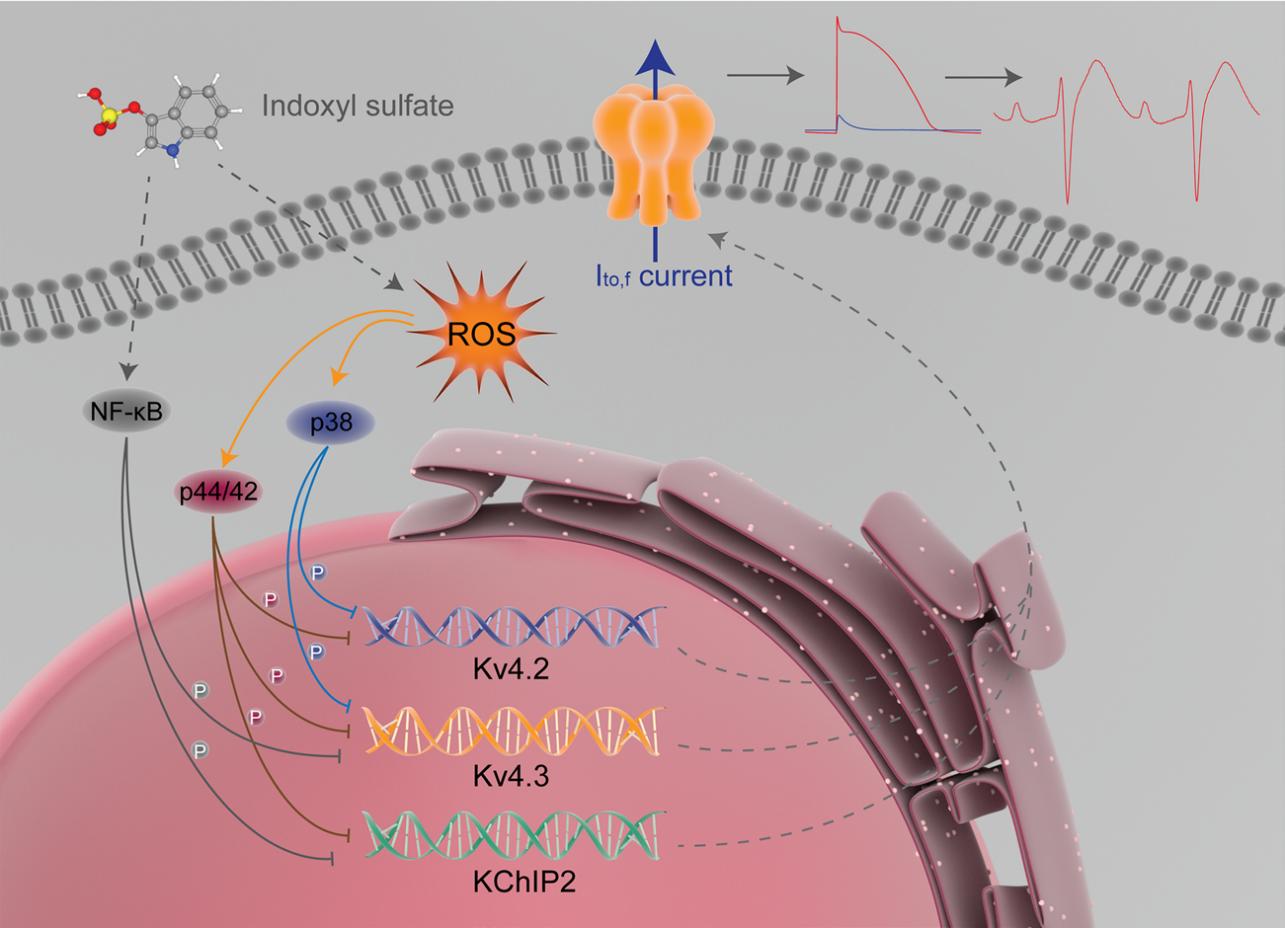
Proposed mechanism for I_to,f_ regulated by IS. IS reduces the expression of I_to,f_-related proteins by activating ROS/p38 MAPK, ROS–p44/42 MAPK, and NF-κB signaling pathways. Finally, I_to,f_ is downregulated, and the action potential duration and QT interval changes accordingly, which contributes to increasing the susceptibility to arrhythmia in CKD rats.

The p38 MAPK, p44/42 MAPK (Erk1/2), and NF-κB signaling pathways are capable of causing changes of various ion channel currents. The MAPK signaling pathway can regulate voltage-gated K^+^ channels in rat coronary arterial smooth muscle cells, which is involved in ethanol-induced coronary artery contraction (51). Liu et al. found that leukotriene B_4_ inhibited L-type calcium channels of vascular smooth muscle cells through the p38 signaling pathway (52). Wu et al. found that CXCL13, upregulated by peripheral inflammation, acted on CXCR5 on dorsal root ganglia neurons, and activated p38 MAPK, which increased Na_v_1.8 current density and further helped to maintain inflammatory pain (53). The ERK pathway can mediate the inhibitory effect of nociceptin/orphanin FQ on delayed rectifier potassium currents in acutely isolated rat cerebral parietal cortical neurons (54) and can mediate the sensitization of human transient receptor potential vanilloid 3 channel (55). Chen et al. found that curcumin enhanced the expression of large conductance Ca^2+^-activated potassium channels protein by inhibiting proteasome degradation and activating ERK signaling pathway (56). NF-κB is involved in vascular large conductance Ca^2+^-activated potassium channel dysfunction promoted by glucose fluctuations (57) and a valsartan-induced increase in KIR2.1 expression in myocardial infarction rats (58). A study conducted by Liu et al. shows that advanced glycation end products reduced Kv1.2/1.5 expression and inhibited Kv currents by activating the NF-κB signaling pathway (59). Mitrokhin et al. found that IL-2–induced phosphorylation of NF-κB upregulated the nonselective conductance in human cardiac fibroblast (60). Because p38 MAPK, p44/42 MAPK, and NF-κB signaling pathways can regulate the changes of some ion channel current, and p38 MAPK and p44/42 MAPK are the main downstream signaling pathways of ROS in some pathological processes (32, 33), we speculated that IS might regulate p38 MAPK, p44/42 MAPK, and NF-κB signaling pathways through ROS production and further cause the changes of I_to,f_-related proteins and I_to,f_ densities. The main proteins of p38 MAPK, p44/42 MAPK, and NF-κB signaling pathways were detected by Western blot. We found that IS increased the phosphorylation of p38 MAPK, p44/42 MAPK, and NF-κB both in vivo and in vitro. NAC could partially reverse the phosphorylation of p38 MAPK and p44/42 MAPK, but NAC has no effect on the phosphorylation of NF-κB. Pretreatment with SB203580, a p38MAPK inhibitor; U0126-EtOH, a p44/42 MAPK inhibitor; or BAY11-7082, an NF-κB inhibitor, significantly inhibited IS-induced p38MAPK, p44/42 MAPK, and NF-κB activation. In addition, SB203580 ameliorated the suppression of Kv4.2 and Kv4.3 proteins caused by IS, U0126-EtOH ameliorated IS-induced reductions in Kv4.2, Kv4.3, and KChIP2 proteins, and BAY11-7082 partially reversed the downregulation of Kv4.3 and KChIP2 proteins caused by IS. These signaling pathway inhibitors also ameliorated the downregulation of I_to,f_ density, accelerated activation and decay of I_to,f_, and prolonged AP. This suggests that IS can primarily reduce the expressions of Kv4.2 and Kv4.3 proteins by activating the ROS/p38 MAPK signaling pathway; Kv4.2, Kv4.3, and KChIP2 proteins by activating ROS–p44/42 MAPK signaling pathway; and Kv4.3 and KChIP2 proteins by activating the NF-κB signaling pathway. Finally, the I_to,f_ is downregulated, and the APD and QT interval changes accordingly, which can increase the susceptibility to ventricular arrhythmia in CKD rats (Figure 9). 

There are some limitations. First, the electrophysiological data of isolated adult cardiomyocytes are lacking, but we have used NRVMs for patch clamp experiments, which can already demonstrate the electrophysiological mechanism related to IS. Second, the clinical application is limited due to the lack of clinical case data to further verify the relationship between I_to,f_ and CKD-related arrhythmia. Third, CKD-related arrhythmia may involve changes in various ion channel currents, which requires further research in the future.

In summary, our results reveal a new possible mechanism for CKD-related ventricular arrhythmia. These data suggest that IS reduces I_to,f_-related proteins and I_to,f_ densities by activating ROS/MAPK and NF-κB signaling pathways, and the APD and QT interval are subsequently prolonged, which contributes to increasing the susceptibility to arrhythmia in CKD.

## Methods

### Animals.

Eight-week-old and 1- to 2-day-old male Wistar rats were purchased from the laboratory animal center of Soochow University. The rat CKD model was created as previously described (61, 62). Briefly, 8-week-old male Wistar rats were first inflicted with 2/3 electrocoagulation of the left renal cortex and then underwent right total nephrectomy 1 week later. The sham group was simultaneously generated through sham surgery on normal male Wistar rats. In the CKD plus BB536 group, BB536 — a bifidobacterium longum strain that has been confirmed to have the potential to reduce serum IS levels (31) — was utilized, and CKD rats in this group were fed a diet containing 1% BB536 (Morinaga; Morinaga Milk) for 8 weeks after right nephrectomy. Wistar rats of the IS treatment group were given IS (Cayman Chemical) at a single i.p. injection dose of 100 mg/kg daily for 8 weeks. Normal rats in the vehicle group were given an equal volume of phosphate-buffered saline (PBS) daily by i.p. injection. All rats were housed under controlled room temperature and appropriate humidity with 12-hour light/dark cycle and had free access to water and food. At the end of the experiment, all rats were euthanized (by i.p. injection of pentobarbital, 12 mg/kg); the blood samples and heart tissue of the rats were collected for subsequent experiments.

### Isolation and culture of NRVMs.

NRVMs were isolated from 1- to 2-day-old male Wistar rats as previously described (63). In brief, their hearts were taken out and the lower two-thirds of the ventricles were minced into small pieces in PBS. The ventricular debris was continuously digested in a solution containing 0.06% trypsin (MilliporeSigma) and 81 μg/mL pancreatin (MilliporeSigma) at 37°C. The supernatant of each step was added to DMEM (Thermo Fisher Scientific) containing 10% FBS (Thermo Fisher Scientific) and centrifuged at 395*g* for 10 minutes at room temperature, the cell pellet was resuspended in DMEM containing 10% FBS and 1% penicillin-streptomycin, and then differential preplating for 1.5 hours was used to reduce the presence of noncardiomyocytes. Nonadherent cells were resuspended in DMEM containing 10% FBS, 1% penicillin-streptomycin (Sangon Biotech), and 0.1 mmol/L bromodeoxyuridine (MilliporeSigma) to inhibit fibroblast proliferation. The medium was changed after 24 hours, and NRVMs were cultured in an incubator at 37°C with 5% CO_2_. All experiments were performed after 3–5 days in culture. To study the effects of IS in NRVMs, they were treated with IS at different concentrations (0, 0.1, 1, 10, and 100 mM) for 24 hours. In addition, NRVMs were pretreated with a ROS scavenger (5 mM N-acetylcysteine, Selleck) for 1 hour prior to and throughout the application of IS in order to determine whether the productions of ROS were involved to the reductions of IS-induced I_to,f_-related protein levels, mRNA levels, and current densities. NRVMs were also pretreated with different NADPH oxidase inhibitors (10 μM DPI, Selleck; 100 μM APO, Selleck) for 1 hour prior to and throughout the application of IS in order to discover the potential source of ROS. To determine whether the activation of p38MAPK, p44/42 MAPK, or NF-κB contributed to the inhibitions of I_to,f_-related protein expression, mRNA expression, and current densities caused by IS, NRVMs were pretreated with a p38MAPK inhibitor (1 μM SB203580, Selleck), a p44/42 MAPK inhibitor (10 μM U0126-EtOH, Selleck), or an NF-κB inhibitor (1 μM BAY 11-7082, Selleck) for 2 hours before and during IS exposure.

### Electrophysiological recording.

Electrophysiological recordings of I_to,f_ and APs were performed under the whole-cell patch clamp configuration with an Axopatch 200B amplifier(Axon Instruments; Molecular Devices) as previously described (64).The glass microelectrode was pulled to generate a resistance of 3–5 MΩ; the pipette offset was corrected to zero before forming a Gigaohm (GΩ) seal. In addition, the pipette capacitance, series resistance, and membrane capacitance were compensated in the experiment. The whole-cell current is digitized at 10 kHz and filtered at 2 kHz. All experiments were conducted at room temperature.

To measure the I_to,f_, NRVMs were superfused with modified Tyrode solution of the following composition (in mM): 140 NaCl, 4 KCl, 2 CaCl_2_, 1 MgCl_2_·6 H_2_O, 10 HEPES, 10 D-Glucose, and 0.5 CdCl_2_·2.5 H_2_O (pH 7.4 with NaOH). The pipette solution contained (in mM) 140 KCl, 1 MgCl_2_·6 H_2_O, 10 EGTA, 10 HEPES, and 5 MgATP (pH 7.25 with KOH). I_to,f_ was recorded with a 150 ms step to –80 mV, from a holding potential of –20 mV, followed by a 50 ms prepulse to –40 mV (to discharge Na^+^ current), followed by a series of 500 ms depolarizing steps from +60 to –40 mV. I_to,slow_ was not inspected in this experiment. The amplitude of I_to,f_ was measured as the difference between the initial peak value of I_to,f_ and the residual current value at the end of the depolarization step.

The activation curve of I_to,f_ was fitted by the Boltzmann equation: I/I_max_ = 1 – (1 + exp [(V_m_ – V_0.5_)/k])^–1^, where I/I max is the relative current, V_m_ is the membrane voltage, V_0.5_ is the voltage at half maximum activation, and k is the slope factor. The decay curve of the I_to,f_ was fitted by a monoexponential equation: I = A exp (–t/τ) + C, where t is the decay time at +60 mV, τ is the time constant, A is the amplitude, and C is constant.

AP was recorded using the whole-cell current-clamp as previously described (65). The bath solution used to record the AP contained (in mM) 137 NaCl, 5.4 KCl, 1.8 CaCl_2_, 1 MgCl_2_·6 H_2_O, 10 HEPES, and 0.33 NaH_2_PO_4_·12 H_2_O (pH 7.4 with NaOH), the pipette solution included (in mM) 5 Na_2_ATP, 120 KCl, 1 CaCl_2_, 5 MgCL_2_·6 H_2_O, 11 EGTA, 10 HEPES, and 11 D-Glucose (pH 7.3 with KOH). AP was induced at a frequency of 1Hz.

### Computational modeling.

The computational modeling of AP was further used to investigate the relationship between the reduction of the I_to,f_ caused by IS and AP. The simulation in this study was conducted on the virtual heart page based on http://dev1.thevirtualheart.org/, in order to simulate the change of IS to I_to,f_, we reduced the I_to,f_ amplitude by 50% in the model and completed the stimulation during continuous pacing at a cycle length of 300 ms (S1-S1 interval). The last AP trace in the simulation was recorded and analyzed.

### Electrocardiography.

The electrocardiography was recorded using a limb lead II configuration in the RM6240B/C physiological signal acquisition and processing system (Chengdu Instrument Factory). The QT interval was measured from the Q wave to the end of the T wave and corrected using the Fridericia’s formula (66), 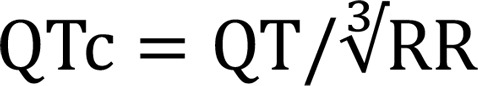. To test the susceptibility to arrhythmia, rats were i.p. injected with isoproterenol (2 mg/kg, Aladdin) and caffeine (120 mg/kg, MilliporeSigma) (67). Arrhythmia was scored based on the following points system: no arrhythmias, 0 points; premature atrial or ventricular beats, 1 point; supraventricular tachycardia or paired premature ventricular beats, 2 points; bigeminal or trigeminal premature ventricular contractions (PVCs) or nonsustained ventricular tachycardia (≥3 but <10 consecutive PVCs), 3 points; and sustained ventricular tachycardia (≥10 consecutive PVCs), 4 points (68).

### RNA isolation and qPCR.

Total RNA isolation and qPCR were performed as previously described (69). Briefly, total RNA was extracted from NRVMs using TRIzol reagent (Invitrogen). mRNA was reverse transcribed into cDNA using the First Strand cDNA Synthesis Kit (Thermo Scientific) at a temperature of 25°C for 5 minutes, 42°C for 60 minutes, and 70°C for 5 minutes. qPCR was performed using SYBR Select Master Mix (Applied Biosystems) in 7500 Fast Real-Time PCR Systems (Applied Biosystems; Thermo Fisher Scientific). The thermal cycler program included the following 3 stages: 95°C for 30 seconds; then, 40 cycles of 95°C for 5 seconds and 60°C for 34 seconds. The last dissociation step was performed at 95°C for 15 seconds, 60°C for 1 minute, and 95°C for 15 seconds. The primer sequences used for PCR were as follows: GAPDH forward, 5′-GACATGCCGCCTGGAGAAAC-3′, and reverse, 5′-AGCCCAGGATGCCCTTTAGT-3′; Kv4.2 forward, 5′-AGGACGCTCTAATTGTGCTGAACG-3′, and reverse, 5′-GTGCGGTAGAAGTTGAGGATGTGG-3′; Kv4.3 forward, 5′-CCTCCGCCAGCAAGTTCACAAG-3′, and reverse, 5′-TGACCAGGACGCCGCTTAGG-3′; and KChIP2 forward, 5′-TACCGAGGCTTCAAGAACGAATGC-3′, and reverse, 5′-CAGAGCCATCGTGGTTGGTGTC-3′. Target gene mRNA expression was normalized to internal control GAPDH. The relative mRNA level was quantified by 2^–ΔΔCt^ method (70), and all experiments were repeated 3 times.

### Western blot analysis.

Western blot was performed as previously mentioned (71). Briefly, the cultured NRVMs and heart tissue were lysed in RIPA lysis buffer (Beyotime) containing 1 mM phenylmethylsulfonyl fluoride (PMSF) (Solarbio). The supernatant was collected, and the protein concentration was determined by the BCA protein assay Kit (Solarbio). Equal amounts of protein were loaded and separated on 10% SDS-PAGE and then transferred to polyvinylidene difluoride membranes. The membranes were blocked with 5% BSA solution at room temperature for 2 hours and incubated with corresponding primary antibodies including anti-GAPDH antibody (1:1000, ab8245, Cell Signaling Technology), anti-Kv4.2 antibody (1:1000, 21298-1-AP, Proteintech), anti-KCND3 (Kv4.3) antibody (1:1000, A6927, ABclonal), anti-KChIP2 antibody (1:1000, ab88542, Abcam), anti–phospho-p38 MAPK antibody (1:1000, 4511T, Cell Signaling Technology), anti–phospho-p44/42 MAPK (Erk1/2) antibody (1:2000, 4370T, Cell Signaling Technology), anti–phospho-NF-κB p65 antibody (1:1000, 3033S, Cell Signaling Technology), anti–p38 MAPK antibody (1:1000, 8690T, Cell Signaling Technology), anti–p44/42 MAPK (Erk1/2) antibody (1:1000, 4695T, Cell Signaling Technology), anti–NF-κB p65 antibody (1:1000, 8242S, Cell Signaling Technology), and NOX2 (1:5000, ab129068, Abcam) at 4°C overnight. The membranes were washed 3 times with tris-buffered saline with Tween 20 (TBST) and then incubated with the appropriate anti–rabbit HRP antibody (7074S, Cell Signaling Technology) or anti–mouse HRP antibody (7076S, Cell Signaling Technology) at room temperature for 2 hours. The membranes were washed again 3 times in TBST. SignalFire Plus ECL Reagent (Cell Signaling Technology) was used to visualize the signal, and the signal was captured by the ChemiDoc XRS+ imaging system (Bio-Rad). Blots were analyzed in the ImageJ 1.47v software (NIH) and normalized to GAPDH.

### Immunofluorescence.

NRVMs were first washed 3 times with PBS for 5 minutes each time, and they were then fixed with 4% paraformaldehyde for 30 minutes at room temperature. The cells were washed again with PBS 3 times for 5 minutes each time and permeated with 0.5% Triton X-100 for 15 minutes. In total, 3% BSA solution was used to block the cells for 30 minutes after they were washed with PBS at room temperature. The cells were then incubated with primary antibodies including anti-cTNI antibody (1:100, 21652-1-AP, Proteintech), anti-Kv4.2 antibody (1:100, 21298-1-AP, Proteintech), anti-KChIP2 antibody (1:100, ab88542, Abcam), and anti–NF-κB p65 antibody (1:400, 8242S, Cell Signaling Technology) at 4°C overnight. Afterward, the cells were washed 3 times with PBS for 5 minutes each time and incubated with goat anti–rabbit IgG (H+L), F(ab’)2 Fragment (Alexa Fluor 594 Conjugate, 8889S, Cell Signaling Technology), or goat anti–mouse IgG (H+L), F(ab’)2 Fragment (Alexa Fluor® 488 Conjugate, 4408S, Cell Signaling Technology), at room temperature in the dark for 1 hour. Three to 4 drops of Mounting Medium with DAPI were applied to stained nuclei after the cells were washed with PBS. Finally, the cells were washed 3 times with PBS for 5 minutes each time and observed using a fluorescence microscope (Olympus). In some cases, confocal laser scanning microscope (Zeiss) was also used to observe the transfer of NF-κB into the nucleus from cytoplasm.

### IHC.

The IHC process was performed as previously reported (72). Briefly, heart tissue samples were fixed with 4% paraformaldehyde (Beyotime) and embedded in paraffin, and 4 μm–thick paraffin sections were subsequently obtained. The sections were deparaffinized and rehydrated, and then the processes of repairing antigen and blocking endogenous peroxidase were carried out. The sections were blocked with 3% BSA at room temperature for 30 minutes. Then, the sections were incubated with anti-Kv4.2 antibody (1:100, 21298-1-AP, Proteintech) and anti-KCND3 (Kv4.3) antibody (1:100, A6927, ABclonal) at 4°C overnight. They were then treated with SignalStain Boost IHC Detection Reagent (HRP, Rabbit) and SignalStain DAB Substrate Kit (Cell Signaling Technology). Finally, all sections were counterstained with hematoxylin, dehydrated, and mounted; the stained sections were observed under a light microscope.

### Detection of ROS.

The in situ levels of ROS in rat hearts were detected by oxidative fluorescent probe DHE (MilliporeSigma) as described previously (73). Briefly, the hearts were embedded in OCT and cut into 8 μm –thick cryostat sections, and the sections were subsequently incubated with DHE (10 μmol/L) in the dark at 37°C for 30 minutes. Intracellular ROS levels were first measured using flow cytometry. Briefly, the single-cell suspension was obtained after NRVMs were digested with trypsin; NRVMs were washed 3 times with PBS and incubated with the peroxide-sensitive dye 2′,7′-Dichlorofluorescein diacetate (H2DCFDA) (10 μmol/L, MilliporeSigma) for 30 minutes at 37°C protected from light. After washing, NRVMs were centrifuged at 395*g* for 10 minutes at room temperature and resuspended in 500 μL PBS. Intracellular ROS levels were also assayed by immunofluorescence as reported previously (74). The culture mediums were removed, and NRVMs were washed 3 times with PBS. NRVMs were incubated with H2DCFDA (10 μmol/L) in the dark at 37°C for 30 minutes, and the cells were washed with PBS again for subsequent detection. Finally, heart sections and cells were detected by fluorescence microscopy, and the images of heart sections were analyzed by the ImageJ 1.47v software (NIH). For flow cytometry, DCF fluorescence was measured with FL-1 495 nm excitation and 529 nm emission on a FACSAria II flow cytometer and FACSDiva software v 8.0.1(BD Biosciences).

### Measurements of IS, creatinine, and blood urea nitrogen.

Blood samples were collected from the abdominal aorta of rats under anesthesia and centrifuged at 1098*g* for 10 minutes at room temperature to obtain serum to obtain serum. The rat heart tissues were homogenized for the determinations of IS levels in heart tissues based on the ELISA. The detections of IS in serum and heart tissues were performed using a rat IS ELISA Kit (Shanghai JiangLai Industrial Limited By Share Ltd.) according to the manufacturer’s protocol. The creatinine levels in the serum were detected with the creatinine assay Kit (colorimetric method) (Nanjing Jiancheng Bioengineering Research Institute Co. Ltd.) according to the manufacturer’s instructions, and the blood urea nitrogen (BUN) levels in the serum were detected with the BUN assay Kit (colorimetric method) (Nanjing Jiancheng Bioengineering Research Institute Co. Ltd.) according to the manufacturer’s instructions.

### Measurement of cell viability.

Cell viability was measured using MTT cell proliferation and cytotoxicity assay kit (Beyotime) according to the manufacturer’s instructions. In Brief, NRVMs were seeded in a 96-well plate with a density of 5 × 10^3^ cells/well. After treatment with different drugs, 10 μL of MTT solution (5 mg/mL) was added to each well. The cells were incubated in the incubator at 37°C for 4 hours. Then, 100 μL of the solution for dissolving formazan was added to each well. Finally, the optical density was measured at 570 nm with a microplate reader (Thermo Fisher Scientific).

### Statistics.

Statistical analysis was performed with OriginPro 2019b software (OriginLab Corporation). Data are presented as mean ± SEM. Comparisons between 2 experimental groups were performed using 2-tailed Student’s *t* test. One-way ANOVA followed by Bonferroni post hoc test was used for comparison of multiple experimental groups. A *P* value of less than 0.05 was considered to be statistically significant.

### Study approval.

All experimental procedures were approved by the Animal Care and Use Committee for Teaching and Research of Soochow University and were performed in accordance with the *Guide for the Care and Use of Laboratory Animals* (National Academies Press, 2011).

## Author contributions

JY designed and conducted all experiments, analyzed data, and wrote the manuscript. HL and CZ analyzed data, and YZ designed animal experiments and corrected manuscript. The authors read and approved the final manuscript.

## Supplementary Material

Supplemental data

## Version 1 02/08/2022

Electronic publication

## Version 2 02/11/2022

The author affiliation address was updated
