# Supplementary material for: Indoxyl sulfate reduces Ito,f by activating ROS/MAPK and NF-κB signaling pathways
Source: JCI Insight. 2022 Feb 8;7(3):e145475. doi: 10.1172/jci.insight.145475 (PMC8855797; doi:10.1172/jci.insight.145475)
Supplement: Supplemental data [file jciinsight-7-145475-s302.pdf]

Supplementary materials

Supplementary Figure 1

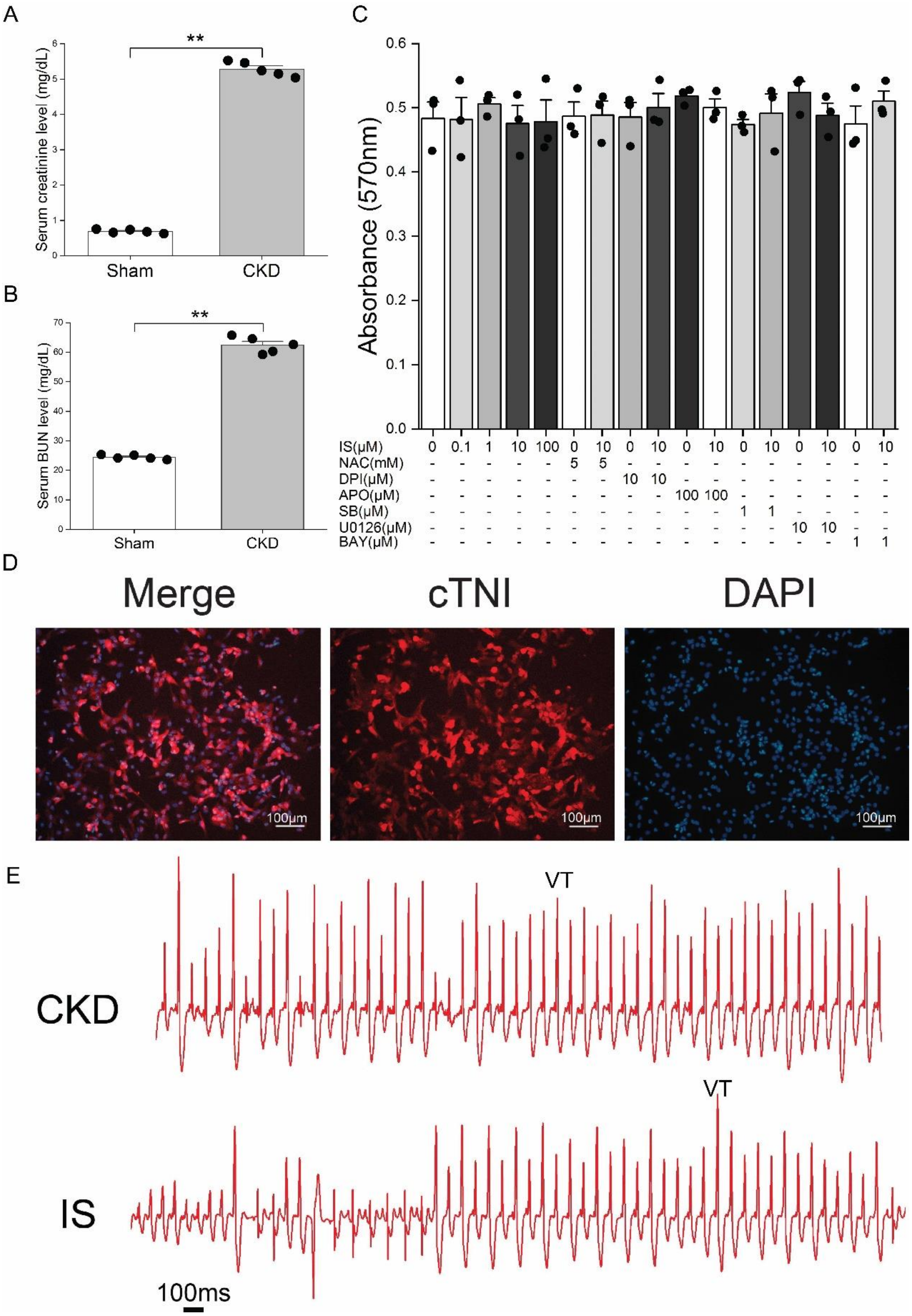

Supplementary Figure 1. Measurements of creatinine, BUN and cell viability, identification of cell purity and amplification

of electrocardiogram strips. (**A**-**B**) Measurements of serum creatinine (**A**) and BUN (**B**) levels in rats (n=5 per group). (**C**)Determination of cell viability based on 3-(4,5-Dimethyl-2-thiazolyl)-2,5-diphenyl-2H-tetrazolium bromide (MTT) assay in NRVMs (n=3 per group). (**D**) Representative fluorescence image of cardiac troponin I (cTNI) in NRVMs. Scale bar,100 μm. (**E**) Enlarged electrocardiogram strips of the rats after the intraperitoneal injection of isoproterenol and caffeine in CKD (n=5) and IS treatment groups (n=6). IS, Indoxyl sulfate; NAC, N-acetylcysteine;DPI, Diphenyleneiodonium chloride;APO,Apocynin;SB, SB203580;U0126, U0126-EtOH;BAY, BAY11-7082;VT,ventricular tachycardia. Data are presented as mean ± SEM. Statistical analysis was performed using two-tailed Student's t-test (**A** and **B**) and one-way ANOVA followed by Bonferroni post-hoc test (**C**). $^{**}P<0.01$.

Supplementary Figure 2

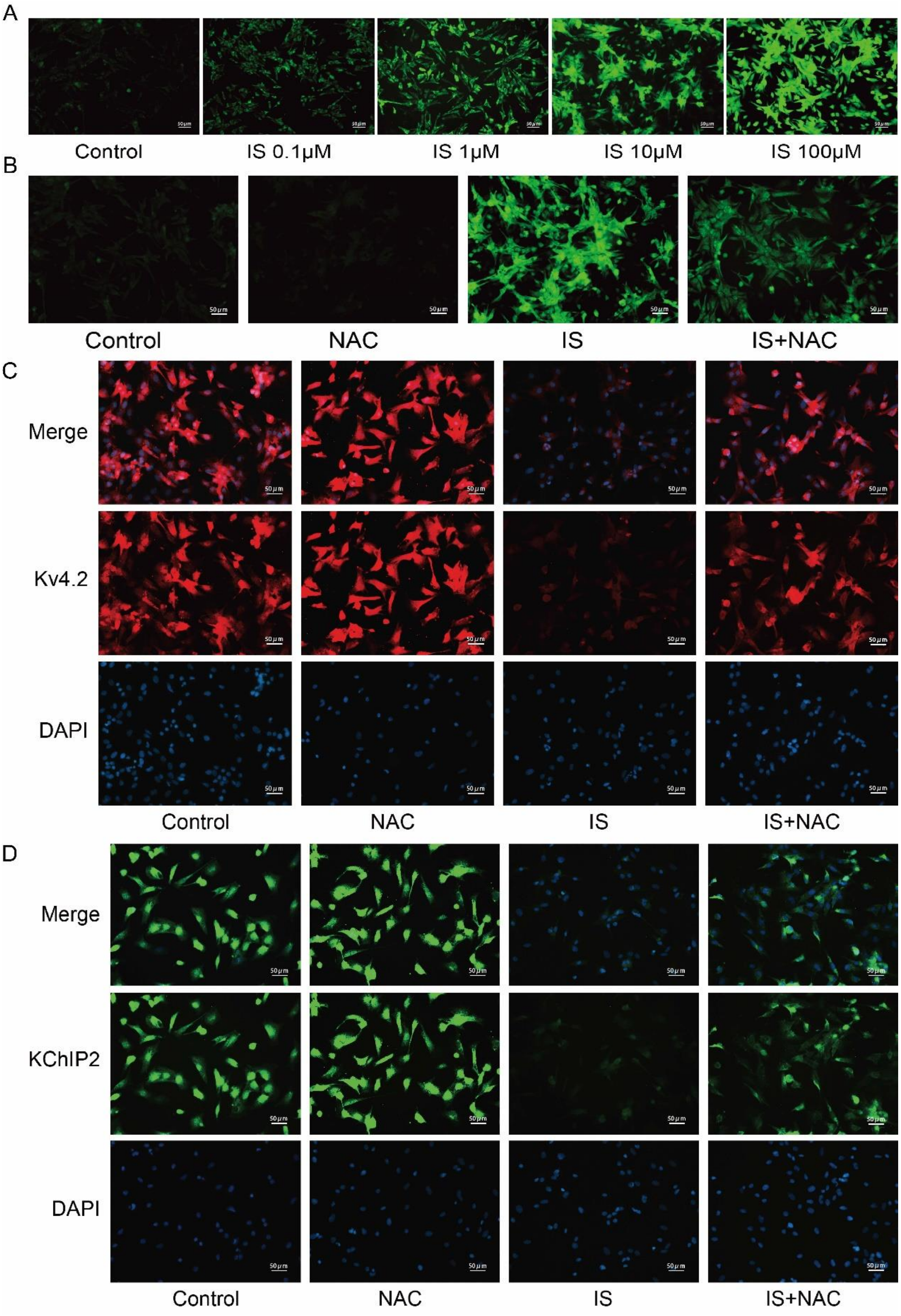

Supplementary Figure 2. Measurements of ROS productions, Kv4.2 proteins and KChIP2 proteins based on Immunofluorescence. (**A**) Representative images of DCF immunofluorescence in NRVMs treated with different concentrations of IS. Scale bar,50 μm. (**B**) Representative images of DCF immunofluorescence in control, NAC, IS and IS+NAC groups. Scale

bar,50 μm. (**C**) Representative fluorescence images of Kv4.2 proteins in control, NAC, IS and IS+NAC groups. Scale bar,50 μm. (**D**) Representative fluorescence images of KChIP2 proteins in control, NAC, IS and IS+NAC groups. Scale bar,50 μm. NAC, N-acetylcysteine.

Supplementary Figure 3

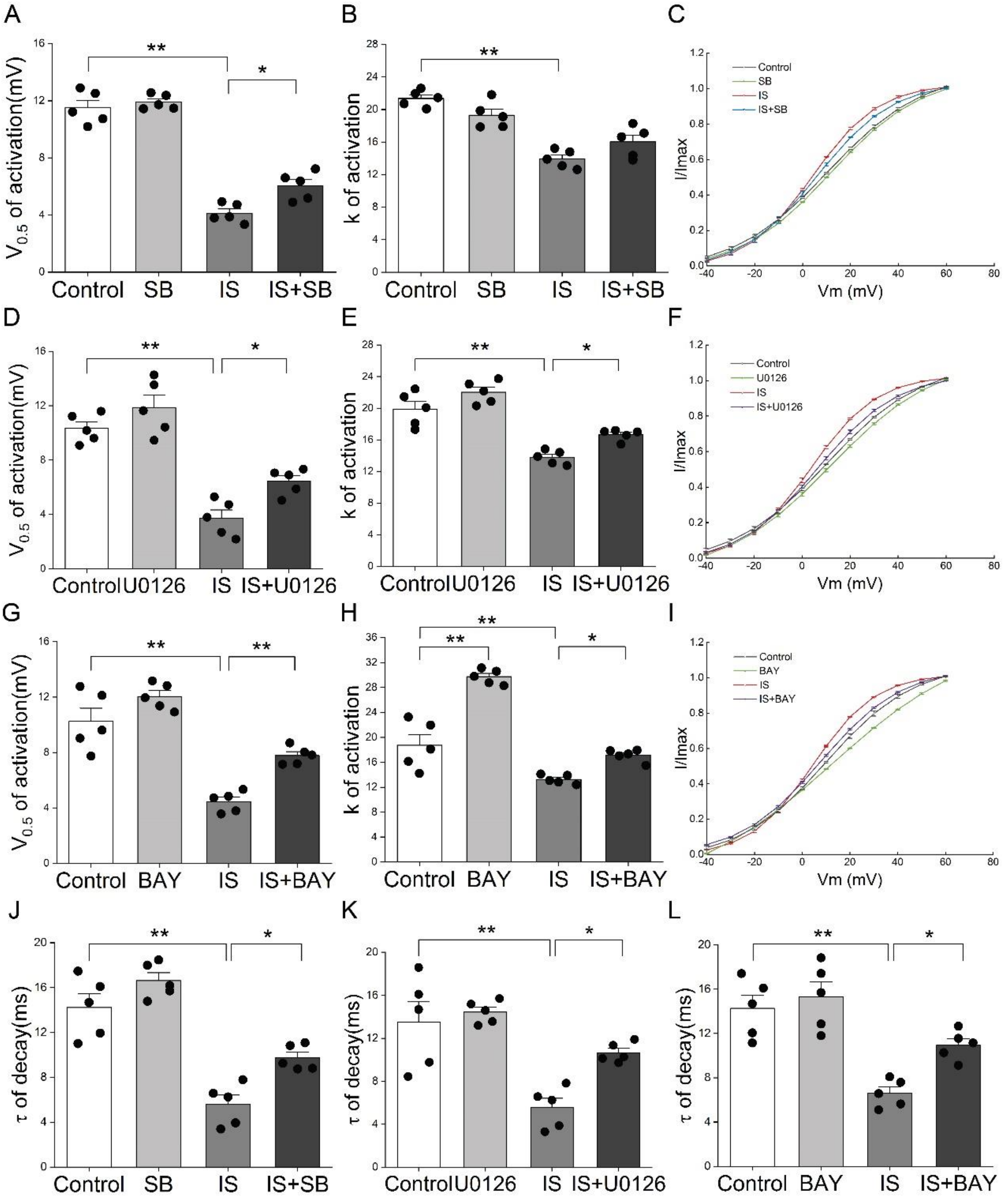

Supplementary Figure 3. IS accelerated the activation and decay of $I_{to,f}$ by activating p38 MAPK, p44/42 MAPK and NF-κB signaling pathway.(**A**-**B**) Average values of half-maximal voltage of activation (V0.5, act) (**A**) and constants(k) of activation(**B**) in control, SB, IS and IS+SB groups (n=5 per group). (**C**) Voltage-dependent activation curves of $I_{to,f}$ in control, SB, IS and IS+SB groups (n=5 per group). (**D**-**E**)Average values of half-maximal voltage of activation (V0.5, act) (**D**) and constants (k) of activation (**E**) in control, U0126, IS and IS+U0126 groups (n=5 per group). (**F**) Voltage-dependent activation curves of $I_{to,f}$ in control, U0126, IS and IS+U0126 groups (n=5 per group). (**G**-**H**) Average values of half-maximal voltage of activation (V0.5, act) (**G**) and constants(k) of activation (**H**) in control, BAY, IS and IS+BAY groups (n=5 per group). (**I**) Voltage-dependent activation curves of $I_{to,f}$ in control, BAY, IS and IS+BAY groups. (**J**-**L**) Average time constants (τ) of decay of $I_{to,f}$ at +60 mV in control, SB, IS and IS+SB groups (**J**), control, U0126, IS and IS+U0126 groups (**K**) and control, BAY, IS and IS+BAY groups (**L**) (n=5 per group). SB, SB203580; U0126, U0126-EtOH; BAY, BAY11-7082. Data are presented as mean ± SEM. Statistical analysis was performed using one-way ANOVA followed by Bonferroni post-hoc test. $^{*}P<0.05$, $^{**}P<0.01$.
